# Low-coordinated copper facilitates the *CH_2_CO affinity at enhanced rectifying interface of Cu/Cu_2_O for efficient CO_2_-to-multicarbon alcohols conversion

**DOI:** 10.1038/s41467-024-49247-4

**Published:** 2024-06-18

**Authors:** Yangyang Zhang, Yanxu Chen, Xiaowen Wang, Yafei Feng, Zechuan Dai, Mingyu Cheng, Genqiang Zhang

**Affiliations:** Hefei National Research Center for Physical Sciences at the Microscale, CAS Key Laboratory of Materials for Energy Conversion, Department of Materials Science and Engineering, University of Science and Technology of China, Hefei, Anhui China

**Keywords:** Electrocatalysis, Electrocatalysis, Chemical engineering

## Abstract

The carbon−carbon coupling at the Cu/Cu_2_O Schottky interface has been widely recognized as a promising approach for electrocatalytic CO_2_ conversion into value-added alcohols. However, the limited selectivity of C_2+_ alcohols persists due to the insufficient control over rectifying interface characteristics required for precise bonding of oxyhydrocarbons. Herein, we present an investigation into the manipulation of the coordination environment of Cu sites through an in-situ electrochemical reconstruction strategy, which indicates that the construction of low-coordinated Cu sites at the Cu/Cu_2_O interface facilitates the enhanced rectifying interfaces, and induces asymmetric electronic perturbation and faster electron exchange, thereby boosting C-C coupling and bonding oxyhydrocarbons towards the nucleophilic reaction process of *H_2_CCO-CO. Impressively, the low-coordinated Cu sites at the Cu/Cu_2_O interface exhibit superior faradic efficiency of 64.15  ±  1.92% and energy efficiency of ~39.32% for C_2+_ alcohols production, while maintaining stability for over 50 h (faradic efficiency >50%, total current density = 200 mA cm^−2^) in a flow-cell electrolyzer. Theoretical calculations, operando synchrotron radiation Fourier transform infrared spectroscopy, and Raman experiments decipher that the low-coordinated Cu sites at the Cu/Cu_2_O interface can enhance the coverage of *CO and adsorption of *CH_2_CO and CH_2_CHO, facilitating the formation of C_2+_ alcohols.

## Abstract (web summary)

Converting CO_2_ into multicarbon products is highly desirable, but product selectivity needs improvement. Here the authors manipulate the coordination environment of Cu site through in-situ electrochemical reconstruction to enhance CO_2_ conversion to C2+ alcohol.

## Introduction

CO_2_ electrochemical reduction (CER) to produce value-added chemicals and fuel is an available strategy in response to the growing energy and environmental crisis^1^. C_2+_ alcohols are coveted outputs of CER owing to their extensive market potentials and remarkable energy densities^2^. Indubitably, the harmonious cooperation of biphasic Cu/Cu_2_O catalyst stands as an eminent contender in engendering C_2+_ alcohols owing to its heightened predilection towards *CO adsorbates on Cu^+^ and reduced energy barrier for C–C or C_2_–C coupling at the rectifying interface^3,4^. Nevertheless, owing to the precarious stability of oxyhydrocarbons intermediates (wherein, C_2_H_3_O* serves as the watershed of C_2_H_4_ or alcohols) and the oxidation state of Cu, the enduringly biphasic Cu/Cu_2_O catalyst continues to face significant obstacles in suppressing the desorption of C_2_H_3_O* and enhancing the yield of alcohols compared to hydrocarbons in the highly reductive environmental^5^. Therefore, the feasible strategies are demanded to develop the architectural blueprint of catalysts and electrolytic systems for preserving the oxidation state and bolstering the CO_2_ performance of copper-based catalysts, including but not limited to elemental doping^6^, interface engineering^7,8^, intermediate confinement^9^, and pulse CO_2_ electrolysis (P-eCO_2_R owing the straightforward and readily adjustable means of manipulating anodic potentials for facilitating the formation of Cu oxide species)^10,11^. Especially, the Mott−Schottky catalyst possesses the remarkable ability to hinder the accumulation of electrons, thereby safeguarding the integrity of Cu–O bonds, while simultaneously enabling swift electron transfer courtesy of its built-in electric field^12,13^. Therefore, it necessitates the employment of intricate catalyst configuration and fabrication techniques to elevate the rectifying interface effects of Cu/Cu_2_O for the purpose of bonding oxyhydrocarbons.

Several strategies can improve the selectivity of oxyhydrocarbons in CER, including the high concentration of local *CO around the active sites^14,15^, doping modification of copper catalysts with heteroatoms^16,17^, building of crystal defects and low coordination of copper^12,18,19^. Effectively, the coverage of *CO can be facilitated on the low-coordinated Cu sites of oxide-derived Cu, leading to its subsequent hydrogenation into *COH, which is essential for the coupling of OC−COH^20,21^. For example, Liang group reported a fragmented Cu catalyst with abundant low-coordinated sites by electrochemical reconstruction of B-doped Cu_2_O, which exhibited a C_2+_ products faradaic efficiency of 77.8% at 300 mA cm^−2^ (see ref. ^19^). Theoretical computations have demonstrated that the *CO bindings could be strengthened on low-coordinated copper sites and prefer to coupling with *COH instead of *CO dimerization^22^. Moreover, the incorporation of halide species and the creation of oxygen vacancy also contribute to the formation of low-coordinated metal and the controlled generation of intermediates^23,24^. Sun and co-worker intervened the behavior of low-coordination chloride ion (Cl^−^) adsorption on the surface of a silver hollow fiber (Ag HF) electrode in 3 M KCl electrolyte, which resulted in the high concentration of *CO on low-coordination Ag–Cl state for CER^25^. However, only few studies provide insight on the priority of enhanced rectifying interface effects for bonding oxyhydrocarbons on the low-coordinated Cu/Cu_2_O in comparison to the pure Cu/Cu_2_O Mott–Schottky catalyst. Thus, the mechanism exploration of low-coordinated Cu/Cu_2_O on stabilizing *CO and oxyhydrocarbons intermediates is significant.

Herein, we demonstrated a low-coordinated Cu/Cu_2_O Mott–Schottky catalyst with an enhanced rectifying interface (Cu_L_/Cu_2_O). This catalyst adjusts the electron densities through interfacial charge exchange, leveraging the difference in Cu and Cu_2_O work functions, which can effectively form bonds between *CO and oxyhydrocarbons under CER conditions. Furthermore, Cu_L_/Cu_2_O catalyst generated highly selective catalytic sites for the coupling reaction of *CO–COH and hydrogenation of C_2_H_2_O* intermediate to C_2+_ alcohols. Chlorine-doped cuprous oxide (Cl–Cu_2_O) and pure Cu_2_O were selected as the precursors and electrochemically reconstructed to unsaturated-coordinate Cu_L_/Cu_2_O and pure Cu_P_/Cu_2_O. The low-coordinated Cu_L_/Cu_2_O achieved a C_2+_ alcohols faradic efficiency (FE_alcohols_) of 64.15 ± 1.92% with the corresponding energy efficiency of ~39.32%. In addition, a stable C_2+_ alcohols faradaic efficiency of >50% was also obtained during a continuous 50 h chronopotentiometry experiment. Our research provides a platform for the rational design of low-coordinated Cu–Cu_2_O Mott–Schottky catalysts and analyzes the key elements for efficient synthesis of C_2+_ alcohols.

## Results

### Synthesis and characterization of Cu_L_/Cu_2_O nanoparticles

**Fig. 1:**
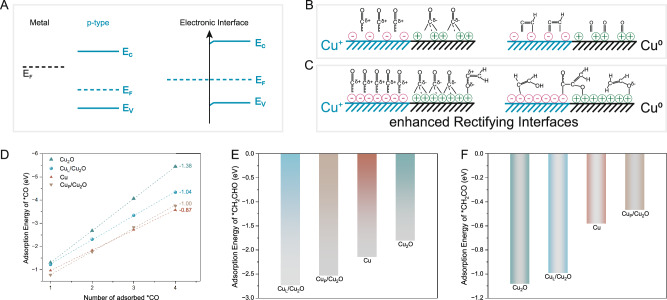
Catalytic mechanism of CuL/Cu2O and free energy of intermediates. **A** Schematic diagram of rectifying interfaces in Mott–Schottky catalysts, where E_F_, E_C_, E_V_ are Fermi levels, conduction band and valence band of semiconductors, respectively. **B** The adsorption of intermediates and C–C coupling reaction on Cu_P_/Cu_2_O. **C** The adsorption of intermediates and C–C or C_2_–C coupling reaction on Cu_L_/Cu_2_O. **D** Free energy versus the number of adsorbed *CO intermediates on the catalyst models. **E** The formation energy of *CH_2_CHO on Cu, Cu_2_O, Cu_P_/Cu_2_O, and Cu_L_/Cu_2_O. **F** The formation energy of *CH_2_CO on Cu, Cu_2_O, Cu_P_/Cu_2_O, and Cu_L_/Cu_2_O.

The Cu_L_/Cu_2_O catalysts were synthesized using a simple electrochemical reconstruction strategy on Cl–Cu_2_O nanoparticles (see details in “Methods”, Supplementary Figs. 1–4). The Cu_L_/Cu_2_O catalysts are designed to function as a Mott–Schottky catalyst of Cu/Cu_2_O, which generates enhanced rectifying interfaces between electron-deficient metal and electron-rich region because of the difference in work function (Fig. 1A). The rectifying interface effect of Cu–Cu_2_O Mott–Schottky catalyst adjusts the electronic densities through interfacial charge exchange, resulting in reduced adsorption resistance for intermediates This effect also leads to higher catalytic performance due to the electronic perturbation at both sides of the interface (Fig. 1B)^26^. Enhancement of the rectifying interface effect, however, can be achieved through the smaller size and defects of crystals^27,28^, which contributes to faster electron transfer and a denser asymmetric charge distribution (Fig. 1C). Furthermore, the accumulable electron density indicates that the *d* → 2π* back donation induce the transfer of electron density from Cu to the *CO intermediates^29,30^. The adsorption abilities for intermediates on different catalyst models are quantified by DFT calculation (Supplementary Figs. 5–8 and Supplementary Table 3). Figure 1D demonstrates that all these models exhibit a linear relationship between the adsorption energy and *CO coverage. Notably, the Cu_L_/Cu_2_O model shows a higher stark tuning slope (−1.04), indicating an enhanced interaction between *CO and the catalyst surface, resulting in increased adsorption strength and coverage. In addition, the Cu_2_O model bonds more firmly with CO molecules than other models due to the presence of stepped sites, confirming the affinity for *CO adsorbates on Cu^+^ sites, as stated in previous literature^31,32^. Subsequently, the adsorption energy of CH_2_CHO (a branching intermediate for ethanol or ethylene) and CH_2_CO intermediates were calculated to assess the potential capacities for ethanol and C_3_ products (Fig. 1E, F). Both of them exhibit strong adsorption on the surface of Cu_L_/Cu_2_O model. The *CH_2_CO molecular, with the C(2) end of H_2_C(1)C(2)O possessing a high positive charge, serves as a key intermediate for the C_2_–C coupling reaction, suggesting the C_2_–C coupling as a nucleophilic addition reaction process in the mixed-valence boundary region^33^. Thus, the low-coordinated Cu_L_/Cu_2_O Mott–Schottky catalyst has an enhanced affinity for intermediates compared to pure Cu_P_/Cu_2_O.

**Fig. 2:**
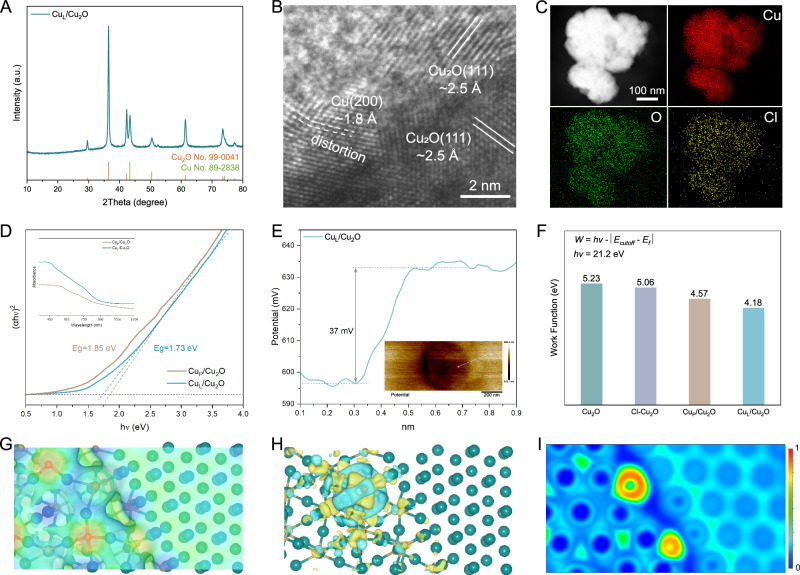
Structures of Cu_L_/Cu_2_O. **A** XRD pattern, **B** HRTEM image, and **C** corresponding elemental mapping of Cu_L_/Cu_2_O showing the interface between Cu and Cu_2_O domains. **D** The Tauc plots (*αℏν*)^2^ versus light energy (*ℏv*) derived by transforming the Kubelka–Munk function on the basis of the inserted UV–vis diffuse absorption spectrum, where Eg is energy bandgap. **E** The surface potential of Cu_L_/Cu_2_O and corresponding variation of surface potential along with the orientation of the white line, where the gray arrows are the difference between the vertical coordinates corresponding to the two dashed lines and the darker color means lower surface potential. **F** Work function determined by UPS measurements. **G** Electrostatic potential, **H** 3D electron density difference distributions with yellow indicating charge density accumulation and blue indicating deplete and **I** 2D display of electron localization function of Cu_L_/Cu_2_O: red displays high electron density and blue is low.

To verify the enhanced rectifying interfaces of Cu_L_/Cu_2_O, a series of experiments were conducted. The sharp peaks of XRD patterns matched perfectly with the standard cubic Cu_2_O (JCPDS No. 99-0041) and Cu (JCPDS No. 89-2838), providing evidence for the coexistence of Cu^+^ and Cu^0^ (Fig. 2A). The SEM and TEM images show that the reconstructed Cu_L_/Cu_2_O nanoparticles exhibit smaller particle sizes ranging from 200 to 300 nm compared to Cu_P_/Cu_2_O, with no serious agglomeration (Supplementary Figs. 9 and 10). From the high-resolution TEM and SAED images of Cu_L_/Cu_2_O (Fig. 2B and Supplementary Fig. 10C), the lattice fringes with interplanar spacing of 0.25 and 0.18 nm are classified to the d spacing of exposed Cu_2_O (111) and Cu (200) planes, respectively. The distortion of Cu(200) may be caused by the potential surface defect owing to the counter diffusion of lattice O from the Cu_2_O/Cu interface with low energy barriers^34^. The distorted Cu(200) can induce a low-coordinated Cu and reduce the barriers of intermediates adsorption^35^. Energy dispersive X-ray spectra (EDS) mapping analysis shows the presence of residual chlorine with a content of 1.14 at% and a homogeneous distribution among Cu, Cl, and O (Fig. 2C, Supplementary Fig. 10D and Supplementary Table S2). The enhanced rectifying interfaces of low-coordinated Cu_L_/Cu_2_O result in an increased absorption edge in the ultraviolet–visible (UV–vis) diffuse reflectance spectrum across the entire wavelength range (inserted in Fig. 2D). Based on the Kubelka−Munk function, the optical bandgap of a semiconductor can be extrapolated from the Tauc plot (the curve of converted (*αhν*)^1/n^ versus *hν* from the UV-vis spectrum, where *α*, *h*, and *ν* are the absorption coefficient, Planck constant, and frequency of the photon, respectively, and *n* is 1/2 for a direct bandgap semiconductor or 2 for an indirect bandgap semiconductor)^33^. The Tauc plot exhibits a well-matched linear fit with *n* = 1/2, which is in line with previous articles supporting Cu_2_O as a direct bandgap semiconductor (Supplementary Fig. 11)^36^. The E_g_ value of Cu_L_/Cu_2_O is calculated to be 1.73 eV by measuring the x-axis intercept of an extrapolated line from the linear regime of the curve, which is smaller than that of Cu_P_/Cu_2_O (1.85 eV). The Cu_L_/Cu_2_O sample with a minimum band energy and highest conductivity is beneficial for enhancing the rectifying interface effect. Kelvin probe force microscopy (KPFM) was carried out to explore the internal electric field and work function of Cu_L_/Cu_2_O (Fig. 2E). The contact potential difference (CPD) profile shows light and dark variations due to the different work functions of sample and substrate^37^. According to the CPD profiles, the work function of Cu_L_/Cu_2_O was defined to be the lowest among the other samples (Supplementary Fig. 12). Figure 2E exhibits the contact potential difference (CPD) profiles for Cu_L_/Cu_2_O, from which an estimate of the average surface potential of 0.037 V can be derived. These results are in accordance with the experimental expectations of Ultraviolet photoelectron spectroscopy (UPS). Importantly, UPS experiments were performed to calculate the work function (WF) of samples for estimating the electron binding factor (Fig. 2F). The experimental WF values for Cu_2_O, Cl-Cu_2_O, Cu_P_/Cu_2_O, and Cu_L_/Cu_2_O, calculated by recording the secondary electron cutoff and Fermi edge (Supplementary Fig. 13), are 5.23, 5.06, 4.57, and 4.18, respectively. These values suggest that Cu_L_/Cu_2_O has a smaller escaping resistance for electrons from the surface, which potentially facilitates a synergistic rectifying interface to enhance the asymmetric adsorption of intermediates. The theoretical electrostatic potential energies of the samples are plotted in Fig. 2G, where the surface potentials of the models are calibrated based on the corresponding Fermi levels (E_Fermi_). Noteworthy, the charge transfer at the interface of Cu_L_/Cu_2_O results in the formation of interface dipole, thereby decreasing the WF of Cu_L_/Cu_2_O in the vacuum region. The distribution of electron density differences of the internal electric field inside Cu_L_/Cu_2_O were explored to estimate the potential adsorption of electrically charged intermediates. As shown in Fig. 2H, the electron clouds around the side of Cu^+^ at the rectifying interface of Cu_L_/Cu_2_O are much more enriched due to the stronger electronegativity of O than Cl and a low-coordination number than that of Cu_P_/Cu_2_O (Supplementary Fig. 14 and Supplementary Table 4), indicating the preferred nucleophilic addition reaction process on Cu_L_/Cu_2_O. Due to the difference in electron density, the Cu^+^ region becomes nucleophilic, while the Cu^0^ region prefers an electrophilic addition reaction process (Fig. 2I). In conclusion, the faster electron exchange at enhanced rectifying interface regions, resulting from the asymmetric electron aggregation, will boost the nucleophilic or electrophilic addition reaction process for C–C or C_2_–C coupling. Generally, the enhanced adsorption of negatively charged *CO may focus on the electrophilic Cu sites, while the C_2_ end of *C_2_H_2_O, with a high positive charge, prefers bonding with nucleophilic Cu_2_O.

**Fig. 3:**
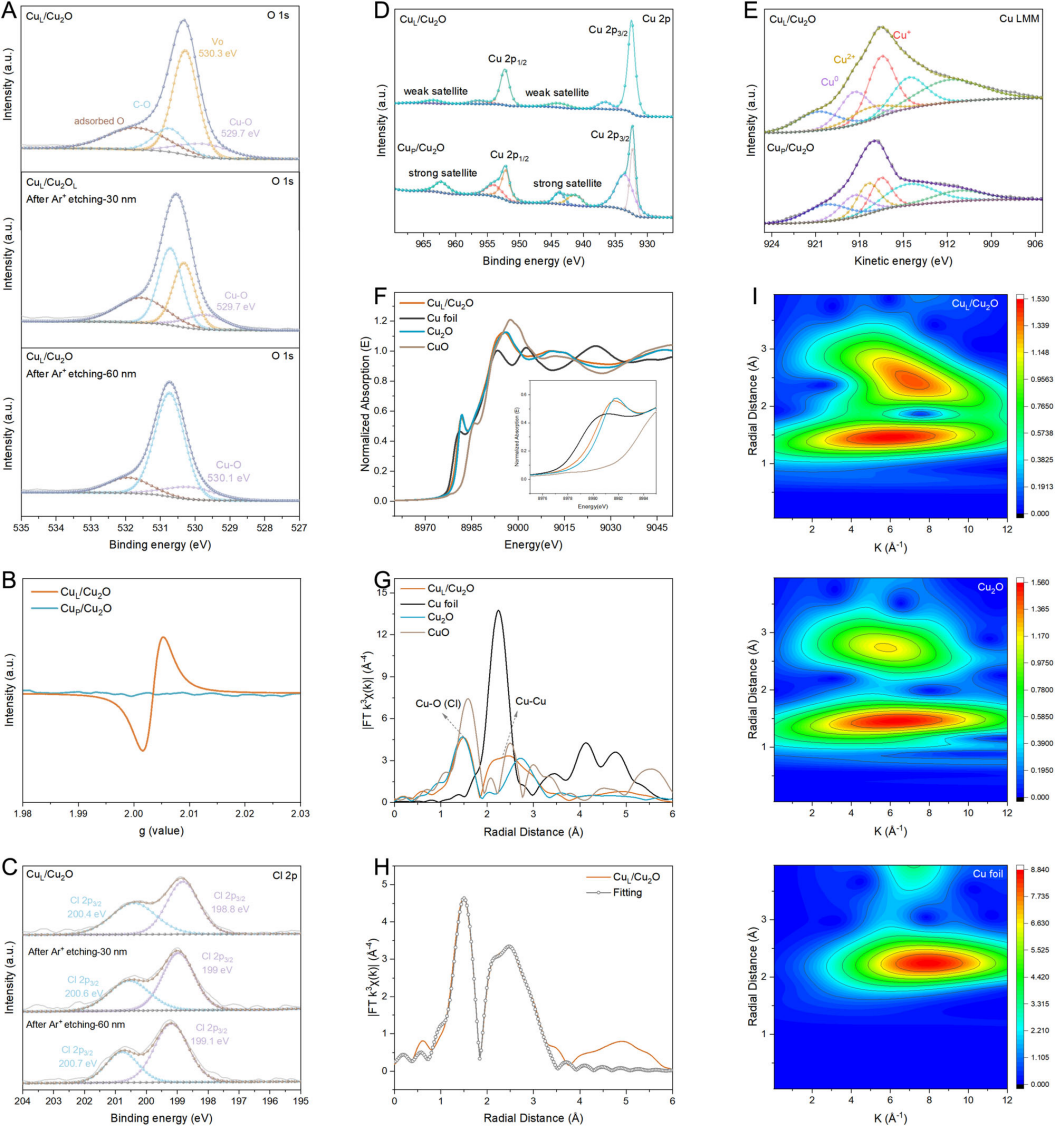
Electron structures and coordination of Cu_L_/Cu_2_O. **A** O *1s* XPS spectra of Cu_L_/Cu_2_O with or without Ar^+^ etching. **B** EPR spectra of Cu_L_/Cu_2_O and Cu_P_/Cu_2_O. **C** Cl *2p*, **D** Cu *2p*, and **E** Cu LMM Auger spectra of Cu_P_/Cu_2_O and Cu_L_/Cu_2_O. **F** The normalized Cu K-edge EXAFS spectra and **G** Fourier transform k^3^-weignted EXAFS for Cu foil, Cu_2_O, CuO, and Cu_L_/Cu_2_O. **H** EXAFS fitting curve of Cu_L_/Cu_2_O. **I** wavelet transform for the k^3^-weignted EXAFS for Cu foil, Cu_2_O, and Cu_L_/Cu_2_O.

To analyze the origins of enhanced rectifying interfaces, electro paramagnetic resonance (EPR), XPS, and X-ray absorption spectroscopy were utilized to characterize the refined structure of Cu_L_/Cu_2_O. As shown in Fig. 3A, the O *1s* XPS spectrum is deconvoluted into four peaks situated at 529.7, 530.3, 530.8, and 531.7 eV, corresponding to the lattice oxygen (Cu–O), oxygen vacancies (Vo), C–O bond and adsorbed oxygen on Cu_L_/Cu_2_O samples, respectively^38^. The ratio of Vo/Cu–O demonstrates a descendant trend with Ar^+^ etching, implying surface and subsurface oxygen deficiency in the oxide-derived Cu. The shift of bonding energy of Cu–O toward a higher level also indicates the disappearance of Vo within the depth of Cu_L_/Cu_2_O. A characteristic sign of Vo is observed at *g* = 2.004 (Fig. 3B)^39^, which supports the electron-rich status of oxygen-deficient Cu^+^. However, the Cu_P_/Cu_2_O exhibits a perfect Mott−Schottky structure without any signs of deficient oxygen (Fig. 3B and Supplementary Fig. 15). From the Cl *2p* spectrums in Fig. 3C, the bonding energy of Cu–Cl also shifts to a higher state with deeper Ar^+^ etching due to the presence of sufficient Cl and O atoms in the depth of Cu_L_/Cu_2_O. The clean rectifying interface regions of Cu_L_/Cu_2_O ensure smooth absorption and desorption, as well as the coupling behavior of oxyhydrocarbon intermediates. In contrast with Cu *2p* spectra of the Cu_P_/Cu_2_O species, the Cu *2p* 3/2 peak of Cu_L_/Cu_2_O shows a strong characteristic signal for Cu^0^/Cu^+^ at 932.3 eV, accompanied by weak satellite peaks (Fig. 3D). The AES of Cu_P_/Cu_2_O and Cu_L_/Cu_2_O were analyzed by Cu LMM Auger spectra to prove the clearer evidence of the valence states of Cu (Fig. 3E). The kinetic energy of the Auger electron transitions of Cu_L_/Cu_2_O corresponding to Cu^+^, Cu^2+^, and Cu^0^ are measured at 916.4, 917.2, and 918.3 eV, respectively, further confirming the coexistence of Cu^+^ and Cu^0^ (Supplementary Fig. 16)^40^. Synchrotron-based X-ray absorption fine spectroscopy was employed to analyze the coordination environment of Cu. The adsorption edge position of Cu_L_/Cu_2_O (8980 eV) is closer to that of Cu_2_O (8983.5 eV) and Cu foil, while it is further away from that of CuO, indicating the presence of integral valence Cu^*δ*+^ (0 <*δ* <1) in the Cu_L_/Cu_2_O catalyst (Fig. 3F and Supplementary Fig. 17)^41^. Figure 3G shows the Fourier transform (FT) extended X-ray absorption fine structure spectra of Cu_L_/Cu_2_O. A strong signal of the Cu–O bond at 1.5 Å can be detected in Cu_L_/Cu_2_O, accompanied by the Cu–Cu bond at 2.32 Å, which also proves the coexistence of Cu^0^ and oxidized Cu^+^. Furthermore, the Cu–O bong length of Cu_L_/Cu_2_O is slightly longer than that of Cu–O in pure Cu_2_O (1.44 Å), implying the presence of Cu–Cl because of the longer Cu–Cl bond compared to Cu–O^42^. Unfortunately, the Cu–Cl sign has fallen out due to the low content of chlorine elements and the shading impact of the strong Cu–O sign. In addition, the Cu^+^ in Cu_L_/Cu_2_O may have a low-coordination number due to the deficiency of oxygen and chlorine. To better quantify the coordination number of Cu, EXAFS fitting was carried out (Fig. 3H and Supplementary Table 4). The experimental k^3^–weight Cu K-edge EXAFS fits perfectly with the calculated R-space fitting model of Cu_L_/Cu_2_O. According to the fitting results, the coordination number of Cu atom of Cu_L_/Cu_2_O (10.3) is smaller than that of Cu_2_O (12.3) and Cu (12), which correspond to the fitting of Cu_2_O and Cu, respectively (Supplementary Fig. 18). The structure of the Mott–Schottky catalyst was further analyzed by wavelet transform of the EXAFS. As shown in Fig. 3I, both Cu_2_O and Cu_L_/Cu_2_O have the K values centered around 6.3 Å^−1^, while the value from Cu_L_/Cu_2_O is located at 7.1, closer to 7.9 Å^−1^ observed from the Cu foil. These analyses confirm that the low-coordinated Cu_L_/Cu_2_O possesses an electron-rich region and abundant defect sites, which receive nucleophilic addition reaction of C–C or C_2_–C coupling in the mixed-valence boundary region to generate ethanol and n-propanol.

### Performance of CER in the flow cell

**Fig. 4:**
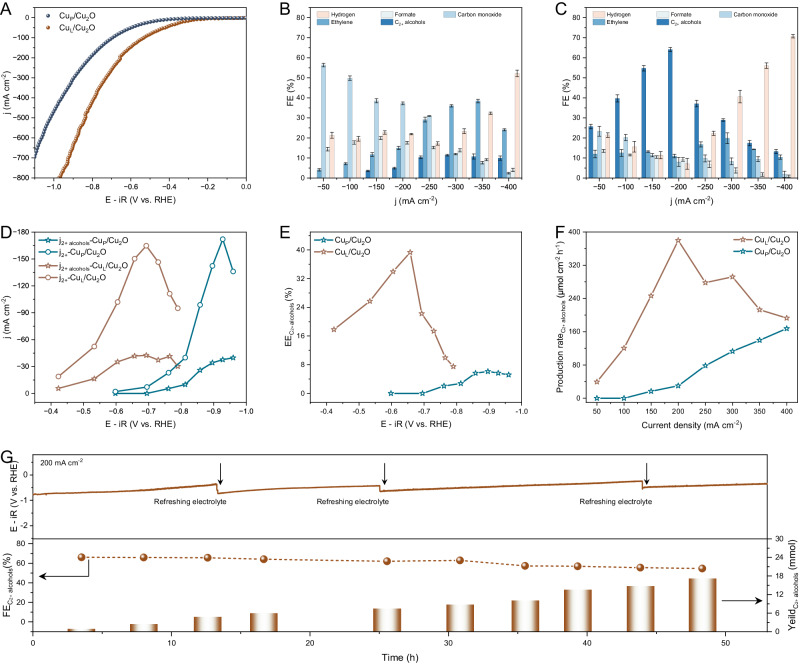
Electrochemical performance of CO_2_ reduction. **A** LSV curves acquired in flow cell using 1 M KOH as electrolyte, where the curves were calibrated by *iR* compensation with a resistance value of 2.65 ± 0.3 Ω. **B**, **C** Faradic efficiencies of products at various j_total_ using Cu_P_/Cu_2_O (**B**) and Cu_L_/Cu_2_O (**C**) catalysts. **D** C_2+_ and C_2+ alcohols_ partial current density of Cu_P_/Cu_2_O and Cu_L_/Cu_2_O catalysts. **E**, **F** Energy efficiencies (**E**) and production rate (**F**) of C_2+ alcohols_ on Cu_P_/Cu_2_O and Cu_L_/Cu_2_O catalysts. **G** Electrochemical stability test at the j_total_ of 200 mA cm^−2^ using the Cu_L_/Cu_2_O catalysts. Error bars show the standard deviations calculated from three independent experiments.

The cathodic electrochemical experiments of CO_2_ reduction reaction were implemented in flow cells using 1 M KOH as electrolyte under ambient conditions (Supplementary Fig. 19). All working potentials are converted into the reversible hydrogen electrode (RHE) scale with *iR* correction of 85%. The linear sweep voltammetry (LSV) curves in Fig. 4A show that the Cu_L_/Cu_2_O catalyst provides higher conductivity and activity for CER, with a total current density (j_total_) of −200 at the applied potential of −0.66 V. The splendid kinetic property of Cu_L_/Cu_2_O reveals a smaller charge-transfer resistance and higher catalytic activity (tafel slope of 232 mV dec^−1^ and electrochemically active surface area of 6.1 mF cm^−2^) for CER (Supplementary Figs. 20 and 21). Remarkably, Cu_L_/Cu_2_O shows excellent selectivity of C_2+_ alcohols with a maximum faradic efficiency (FE) of 64.15 ± 1.92% (ethanol of ~56% and n-propanol of ~8%), whereas Cu_P_/Cu_2_O only reaches a maximum FE_ethylene_ and FE_alcohols_ of ~38.4% and 11.4%, respectively (Fig. 4B, C and Supplementary Fig. 22). As shown in Fig. 4D, at a j_total_ of −200 mA cm^−2^, the partial current density of alcohols (j_alcohols_) rapidly rises to 128.3 mA cm^−2^ on the Cu_L_/Cu_2_O catalyst (the applied potential is −0.66 V), which is 13-fold higher than that of Cu_P_/Cu_2_O (9.9 mA cm^−2^ at −0.81 V). Meanwhile, the half-cell cathodic energy efficiency (EE) and production rate of alcohols on Cu_L_/Cu_2_O also reach optimal values of 39.32% and 379.78 μmol cm^−2^ h^−1^, respectively (Fig. 4E, F). Our results feature one of the most excellent efficiencies for alcohol compared with the reported literature to date (Supplementary Table 5). To further investigate the positive effect of halogen anion on CER, comparative experiments with introducing Cl were conducted. The acid-aqueous solution of NH_4_Cl resulted in the oxidized Cu^2+^ and physically adsorbed Cl on the surface of NH_4_Cl–Cu_2_O (Supplementary Fig. 23). In addition, the distribution of Cl was estimated by XPS after undergoing Ar^+^ etching, confirming the superficial adsorption of Cl (Supplementary Fig. 24 and Supplementary Table 2). The peak values of FE_alcohols_ and j_alcohols_ on NH_4_Cl–Cu_2_O are 17.7% and −50.6 mA cm^−2^, respectively, indicating a slight increase limited by the negligible Cl concentration. However, a significantly enhanced selectivity of alcohols (a maximum FE_alcohols_ of 23.5%) is achieved on Cu_P_/Cu_2_O in 1 M KOH electrolyte with 3 M KCl, suggesting that the specific adsorption of surface-bound Cl, located on the inner Helmholtz planes of Cu electrode, contributes to the C–C or C_2_–C coupling owing to the strong chemical affinity of the anion for meal (Supplementary Fig. 25)^43^. Notably, the Cu_L_/Cu_2_O catalyst still retains its original profile with a mott−schottky interface and unspoiled nanoparticles, as well as an unaltered presence of Cl after CER (Supplementary Figs. 26 and 27). Furthermore, the electrochemical stability of Cu_L_/Cu_2_O for CER was evaluated through chronopotentiometry electrolysis at 200 mA cm^−2^ (Fig. 4G). Despite a steady decline over the course of 50 h electrochemical process, Cu_L_/Cu_2_O achieves a sustained FE_alcohols_ of > 50% and an alcohols yield of 17.1 mmol. However, the mention of pulse electrolysis is necessary for long-term industrial production, which may still be an essential strategy for the CO_2_ electrolysis community, especially due to its unique advantage in electrolysis stability (>1000 h)^44,45^. The structure of Cu_L_/Cu_2_O maintains a classic architecture of Mott–Schottky catalyst, while slight morphological changes occur (Supplementary Fig. 28). Cl–Cu_2_O powders of different particle sizes were prepared and tested under the same electrochemical conditions (Supplementary Figs. 29 and 30). The selectivity of C_2+_ alcohols gradually increases on a gas diffusion electrode loaded with larger-sized particles, which is attributed to the stability of low-coordinated Cu/Cu_2_O. The appropriate particle size contributes to the stabilization of the enhanced rectifying interface of the low-coordinated Cu_L_/Cu_2_O Mott–Schottky catalyst. To explore the effect of electrolytes on the selectivity of C_2+_ alcohols, various electrolytes were used to offer a different pH environment (Supplementary Fig. 31). It undergoes electrochemical reduction reaction of CO_2_ with synergistic effect of CO_2_ coverage and catalyst in pH-compatible electrolyte and thermodynamics-mediated competitive reaction in a strong alkaline electrolyte. The Cl element shows a positive effect on improving the selectivity of C_2+_ products, but the sensitivity of ethylene is stronger than that of C_2+_ alcohols.

### Structure–property correlation and oxidation state of Cu_L_/Cu_2_O

**Fig. 5:**
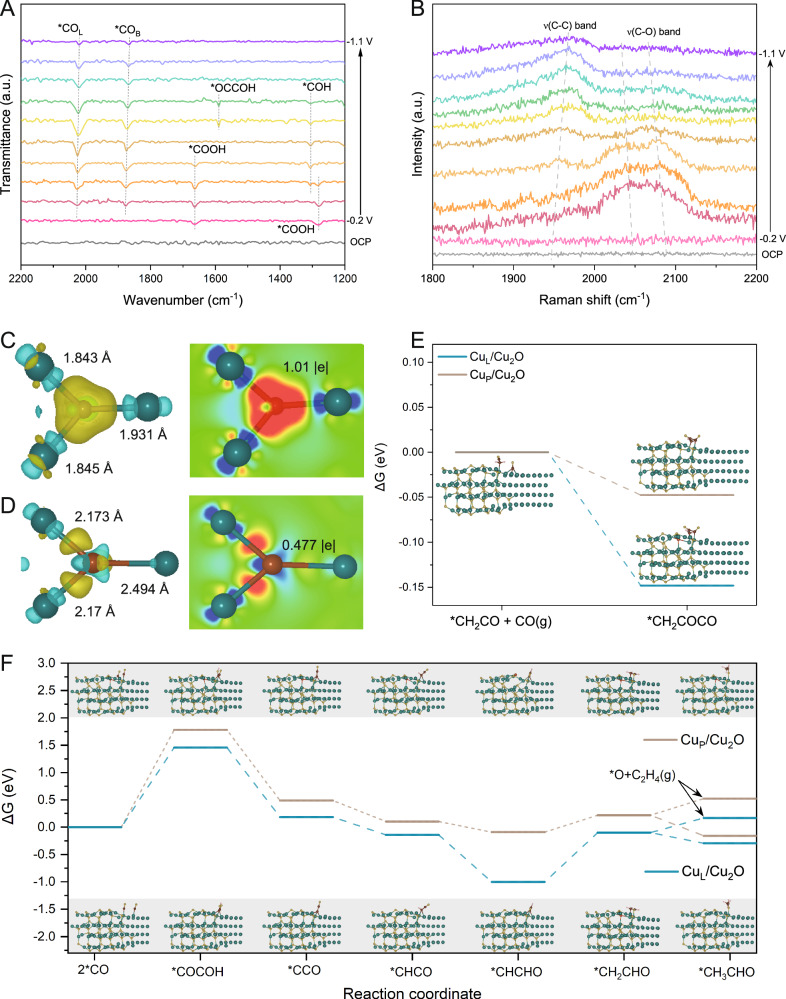
Operando experiments and DFT calculations. **A** Operando FTIR and **B** Raman tests of Cu_L_/Cu_2_O using 0.1 M KHCO_3_ as electrolyte. **C**, **D** Bader charge analysis and bond length of Cu_P_/Cu_2_O and Cu_L_/Cu_2_O, respectively: red and yellow display electron accumulation, blue and cyan indicate electron depletion. **E** Gibbs free energy of *CH_2_COCO intermediates for C_3_ products and **F** C–C coupling to C_2_ products (ethanol and ethylene) on Cu_P_/Cu_2_O and Cu_L_/Cu_2_O catalysts: the steps indicated by the arrows represent the generation of *O and C_2_H_4_.

Operando FTIR and Raman spectroscopy were employed for investigating the reconstruction of catalysts and adsorbates on both Cu_L_/Cu_2_O and Cu_P_/Cu_2_O. The electrochemical reactions were executed in CO_2_-saturated 0.1 M KHCO_3_ electrolyte. The reference spectrum was taken at open-circuit potential, and additional spectra were provided in the range of −0.2 ~ −1.1 V vs. RHE. Two bands emerge at 1663 and 1280 cm^−1^ during CO_2_ bubbling, which corresponds to the C = O and C−OH stretching vibrations of COOH. These peaks indicate the presence of the COOH* intermediate during the reduction of CO_2_ on Cu_L_/Cu_2_O. Furthermore, the C≡O stretch band in the 2000–2100 cm^–1^ range can be attributable to the linear bonding mode of CO molecules (CO_L_)^46^. In contrast to the behavior of the CO_L_ band, the band in the ≈1800–1900 cm^–1^ range, caused by the bridging bonding mode of CO molecules (CO_B_), exhibits a significant degree of hysteresis. The redshift of the CO_B_ band at more negative potentials is a result of the stark tuning effect^47^. Simultaneously with the CO-related band, a band at 1305 cm^−1^ associated with *COH stretching grows, and *COOH disappears in the spectra. which indicates the performance of CO_2_ reduction. The absence of the bands at 1589 cm^−1^ in the transmission spectra points out the possibility of the *OCCOH coupling reaction during the reduction of CO_2_ on Cu_L_/Cu_2_O (Fig. 5A). However, in contrast to Cu_L_/Cu_2_O catalyst, some new signals at 1706, 1543, and 1399 cm^−1^ corresponding to the stretch of *CHO, *OCCO, and *OCHO intermediates emerged in spectra of Cu_P_/Cu_2_O, certifying the coupling mode of OC–CO and possible formate product (Supplementary Fig. 32)^48^. Combining with time-independent spectral analysis (Supplementary Fig. 33A, B), apparent peaks are determined at 1064 and 1558 cm^−1^, which correspond well to the symmetric and asymmetric vibrations of the *OCCOH intermediate, respectively^49,50^. In addition, the stretching vibration at 1335 and 1716 cm^−1^ are in close proximity to the stretching of *OCH_2_CH_3_ and *CHO, respectively, which serve as the important intermediates for the subsequent C–C coupling in the production of C_2+_ alcohols^51,52^. These conclusions are consistent with the results obtained from the product analysis of Cu_P_/Cu_2_O and Cu_L_/Cu_2_O. In the Raman spectra (Fig. 5B and Supplementary Fig. 33D), the Cu_L_/Cu_2_O powder loaded on carbon paper substrate exhibits typical characteristic peaks of adsorbed *CO intermediates, which are deconvoluted into a low-frequency band (LFB) at ∼2045 cm^−1^ and a high-frequency band (HFB) at ∼2097 cm^−1^, which verifies the bonding styles on terrace and step sites, respectively^53,54^. A redshift of the *υ*(CO) band indicates the enhanced interaction between the catalyst surface and *CO, resulting in a higher *CO coverage^55^. In addition, the frequencies of *υ*(CO) bands are correlated with the coordination states of Cu sites. Therefore, the low-coordination states of Cu sites in Cu_L_/Cu_2_O will induce a positive shift of the *d*-band center of Cu and boost the hybridization of the d-band with the 2π* orbital of CO. The HFB modes derived from the low-coordinated Cu sites will favor the breeding of the *υ*(C–C) band (~1960 cm^−1^), where a blueshift may predict the protonation process C–C bonds^29,30^. On the contrary, the weak signs of the *υ*(CO) and *υ*(C–C) bands on Cu_P_/Cu_2_O catalyst also verify the poor *CO coverage and C–C coupling (Supplementary Fig. 34B). To verify the origin of the oxidation state of Cu_L_/Cu_2_O, Raman spectroscopy was conducted in the shift range of 200–1600 cm^−1^, and the results are presented in Supplementary Figs. 33A and 34A. In the case of Cl–Cu_2_O samples, characteristic signals were observed at 224, 425, 522, and 626 cm^−1^, which were attributed to the 2*Γ*^−^_12_, 4*Γ*^−^_12_, *Γ*^+^_25_, and *Γ*^−^_12_ + *Γ*^+^_25_ phonon modes, respectively^56,57^. These Raman signals gradually disappear within the potential range from −0.2 to −0.7 V vs RHE, indicating the complete reduction of Cu_2_O to metallic Cu. In contrast, the Cl–Cu_2_O samples retain the characteristic Raman modes (2*Γ*^−^_12_ and *Γ*^−^_12_ + *Γ*^+^_25_) even at potentials greater than −0.7 V, suggesting that the low-coordinated Cu_L_/Cu_2_O can protect Cu^+^ species against reduction due to a combination of environmental and structural factors. Overall, Cu_L_/Cu_2_O catalyst exhibits a stronger adsorption capacity for *CO due to the restricted rotation and stretching vibration peaks of adsorbed *CO and the Cu–CO bond at around 274 and 358 cm^−1^, respectively^58^. Specifically, the coverage of *CO is closely correlated with the intensity ratio of *ν*(Cu−CO) to *ν*(*CO)^59^. Importantly, the intensity ratio of *ν*(Cu−CO) to *ν*(*CO) of Cu_L_/Cu_2_O is higher in the case of Cu_L_/Cu_2_O compared to Cu_P_/Cu_2_O, implying a greater CO coverage, which is favorable for the oxidation state of Cu^+^. The peaks observed at 1015, 1024, and 1069 cm^−1^ can be assigned to the vibrations of *ν*_1_(C–O) of HCO_3_^−^, OCO antisymmetric stretching from *COOH, and *ν*_2_(C–O) of CO_3_^2−^, respectively^60^. Furthermore, as the potential increases, CO_3_^2−^ accumulates and disappears on the catalyst surface, indicating the dissolution and reduction of CO_2_. Briefly, the origin of the oxidation state of Cu_L_/Cu_2_O can be summarized as follows: (1) the lattice shrinkage resulting from fast electron transfer from Cu to Cu^+^ at the enhanced rectifying interface inhibits the loss of oxygen atoms and stabilizes the chemical valence of the cation Cu^+^ species working with residual Cl atoms^61,62^. (2) The presence of coordination defects and oxygen vacancies increases the adsorption of intermediates rather than forming proton bonds with O atoms of Cu–O to avoid the reduction of Cu^+^ species^63,64^. (3) Strong electronic interactions occur between carbon intermediates and Cu^+^ species^65,66^. When CO forms a hybridization orbital with the Cu *d*-states, the *5σ* and 2π* orbitals of *CO split into bonding and anti-bonding orbitals. In this process, electrons from the Cu d-band are transferred to the *2π* orbitals of *CO through *d*-*2π* back donation, while electrons from the *5σ* orbital of *CO donate to the Cu *d*-band through *5σ*-*d* donation^30^. These effects stabilize the oxidation state of Cu^+^ species during CO_2_ electroreduction, allowing for the synergistic effect of Cu and Cu^+^ species.

The reaction barriers for the hydrogenation process of C_2+_ intermediates on the models of Cu/Cu_2_O with and without Cl, respectively, were calculated by DFT. Firstly, by analyzing the Bader charge and bond length of Cu_L_/Cu_2_O (Fig. 5C, D), it is observed that the lower Bader charge value (0.477 |e|) compared to Cu_P_/Cu_2_O (1.01 |e|) indicates that the populated electronic orbitals of low-coordinated Cu sites will easily bind to intermediates and reduce the rate-determining energy barrier^67^. Cu_L_/Cu_2_O with additionally formed empty orbitals can facilitate electron transfer from hydrogen to nucleophilic intermediates, suggesting the Cu *3d* orbitals overlap with the *π* orbitals of C and further reduced activation energy for CO_2_ hydrogenation on Cu/Cu_2_O^68^. As a result, the nucleophilic addition reaction process of *CH_2_CO–*CO coupling may effectively react on the mixed-valence boundary region^33^, which reduces the formation energy of *CH_2_COCO on Cu_L_/Cu_2_O (−0.148 eV) toward n-propanol (Fig. 5E, Supplementary Fig. 35, and Supplementary Table 6). For the C–C coupling on Cu_L_/Cu_2_O model (Fig. 5F, Supplementary Fig. 36 and Supplementary Table 7), the *CO–COH coupling is the rate-determining step for Cu_L_/Cu_2_O catalyst (1.46 eV), indicating the vital rule of *CO coverage in the post-coupled proton-electron transfer (CPET) reaction. The energy barriers of intermediates gradually decrease until *C_2_H_3_O is reached, which can be further reduced to *C_2_H_4_O or *O for ethanol or C_2_H_4_, respectively. The Cu_L_/Cu_2_O model exhibits a more stable adsorption for C_2_H_4_O* (−0.293 eV) than Cu_P_/Cu_2_O (−0.154 eV), implying facilitated thermodynamics for increasing the adsorption of key intermediate.

## Discussion

The mentioned theoretical and experimental studies provide tangible proof that the enhanced rectifying interface of low-coordinated Cu/Cu_2_O Mott–Schottky catalyst plays an essential role in the selective production of C_2+ alcohols_. We emphasize several elements of the low-coordinated Cu_L_/Cu_2_O catalyst for the upgraded CER performance. Firstly, the fast electron exchange and enhanced intermediate adsorption at synergistic rectifying interface regions are due to the low coordination and asymmetric electron aggregation inside the Cu_L_/Cu_2_O catalyst. In addition, the excellent conductivity and charge difference of Cu^+^/Cu^0^ boost the nucleophilic or electrophilic addition reaction process for C–C or C_2_–C coupling. Secondly, the fast electron transfer from Cu to Cu^+^ at the enhanced rectifying interface can induce lattice shrinkage, inhibiting the loss of residual oxygen atoms responsible for stabilizing the chemical valence of Cu^+^ when working with residual Cl atoms. The clean Cu/Cu_2_O regions ensure smooth absorption and desorption, as well as the coupling behavior of the oxyhydrocarbon intermediates. Thirdly, abundant defects originated from oxygen vacancies and residual Cl, as a result of electrochemically reconstructing Cl–Cu_2_O, supply rich free electrons and landing sites for adsorbed intermediates. These results work together on low-coordinated Cu_L_/Cu_2_O Mott–Schottky catalyst to improve the selectivity of C_2+ alcohols_. The faradic efficiency and energy efficiency of C_2+ alcohols_ are up to 64.15% and 39.32%, respectively, where the stability of over 50 h (FE_C2+ alcohols_>50% at j_total_ = 200 mA cm^−2^) explains the positive effects of the above results.

## Methods

### Synthesis of Cu_P_/Cu_2_O, Cu_L_/Cu_2_O, and NH_4_Cl–Cu_2_O nanoparticles

In the synthesis of the Cu_2_O nanocrystals, glucose served as a reductant, contributing to the reduction process^69^. Detail: a mixed solution containing 2 mL of OA and 5 mL of ethanol was added under magnetic stirring for 30 min after dissolving 1 mmol CuSO_4_·5H_2_O in 15 mL of deionized water and placed in a water bath at 80 °C. Then, 5 mL of 1 M NaOH aqueous solution was added and kept for 10 min. Next, 5 mL of 2 M glucose aqueous solution was added as a reductant to maintain chlorine-free environment for 3 h at 80 °C. The brick red products were obtained by centrifugation and thoroughly cleaned with cyclohexane and ethanol multiple times to eliminate any remaining OA. They were then dried and stored under vacuum at room temperature for future use. As-prepared Cu_2_O nanocrystals (2 mg) were mixed with 20 µL of 5 wt% Nafion in a solution composed of ethanol (190 µL) and water (190 µL). The mixture was deposited onto a gas diffusion layer (2 cm × 2 cm) using an airbrush. It was subsequently electrochemically reduced at –50 mA cm^−2^ for 10 min with flowing CO_2_ in 1 M KOH electrolyte. The obtained catalyst was named as Cu_P_/Cu_2_O, which provided a chlorine-free environment. Using the impregnation approach, a specific quantity of NH_4_Cl was loaded onto the Cu_2_O. In this case, Cu_2_O particles were fully immersed in an NH_4_Cl solution. To reduce acidity and prevent surface etching, a mixed solution of 80% ethanol and 20% water was used as the solvent. The residuals were then promptly dried and stored for later use in a vacuum at room temperature. After electrochemical reconstruction, the obtained catalyst was named NH_4_Cl–Cu_2_O, which exhibited a surface Cl-modified environment.

In addition, 20 mL of water was used to dissolve 2 mmol of Cu(CH_3_COO)_2_·H_2_O. Then, 7 mL of 1  M NaOH aqueous solution was added and kept there while being stirred magnetically for 10 min. After that, 3.5 mL of 2 M NH_2_OH·HCl aqueous solution was added as a reductant and kept there for 1 h. The orange products (Cl–Cu_2_O) were obtained by centrifugation and repeatedly cleaned with methanol and deionized water before being dried and stored for later use under vacuum at room temperature. The Cl–Cu_2_O powders with different particle sizes were prepared by modulating the inputs of reactants and reaction time (15 min–5 h). Similar to the electrochemical reconstruction method described above, the only difference is that Cl–Cu_2_O nanocrystal as the precursor loaded on carbon paper instead of Cu_2_O nanocrystal. The obtained catalyst was named as Cu_L_/Cu_2_O, which showed a chlorine-rich environment.

### Catalyst characterization

X-ray diffraction patterns (XRD) of samples were carried out by TTR-III operating at 40 KV voltage and 15 mA current with Cu Kα radiation (*λ* = 0.15406 nm). Transmission electron microscopy (TEM), high-resolution TEM, high-angle annular darkfield scanning transmission electron microscopy (HAADF-STEM) and energy disperse spectroscopy (EDS) are recorded on a JEM-2100 (JOEL). Scanning electron microscope (SEM) was performed on FESEM SU8200. Raman data were collected on a Renishaw in Via using 785-nm laser. X-ray photoelectron spectroscopy (XPS) data were collected on Kratos Axis supra + . An Ar^+^ beam was employed to detect the quantity of Cl and oxygen vacancy with a electron energy of 12.5 V, filament current of 3 mA, emission current of 7.5 A and energy of 2 KV. UPS excitation source: He light source (*hv* = 21.22 eV), beam spot 2mm. Room temperature UV–Vis absorption was recorded using a Solid 3700 DUV spectrophotometer in the wavelength range of 300–2500 nm. Kelvin probe force microscopy (KPFM) was performed on Atomic force microscopy (AFM) with a Veeco DI Nanoscope MultiMode V system. Electron Paramagnetic Resonance (EPR) was performed on JES-FA200. Fourier transform infrared (FTIR) spectroscopy was performed with a Thermo-Fisher Nicolet iS10. X-ray absorption near edge structure (XANES) and extended X-ray absorption fine structure (EXAFS) data were collected on beamline 14 W at the Shanghai Synchrotron Radiation Facility. The electro-catalysis actions were tested by CorrTest workstations. The gas chromatographs (GC 7900) equipped with a TCD and FID detector is used to detect the generated gas. Liquid NMR were quantified by the Bruker AVANCE III 400MHZ using dimethyl sulfoxide as an internal standard.

### Operando FTIR and Raman tests

Raman experiments were performed using a Renishaw in Via Raman microscope in a commercial flow cell at the excitation laser source of 785 nm, the electrolyte was 0.1 M KHCO_3_ aqueous solution. The Cl-doped Cu_2_O and Cu_2_O precursors were monitored at different potentials and time under the same configuration condition. Then, the intermediates-adsorption measurements were collected signals at different potentials after the precursors were reduced to obtain the Cu_P_/Cu_2_O and Cu_L_/Cu_2_O. The Raman spectra were collected every 0.1 V at a range of −0.2 ~ −1.1 V (vs. RHE) and 150s with bubbling CO_2_ into the electrolyte. FTIR were measured using a Thermo Scientific Nicolet iS50 FTIR Spectrometer with a Pike VeeMAX III attachment. In total, 2 mg of the catalyst was dispersed in a mixture of 0.38 mL ethanol, and 20 μL 5 wt.% Nafion solution (Sigma-Aldrich) and sonicated, then dropped on carbon paper. Spectra were recorded at different potentials and time in a CO_2_–saturated 0.1 M KHCO_3_-D_2_O electrolyte. During the Operando FTIR tests, spectra were collected every 0.1 V and 120s with bubbling CO_2_ into the electrolyte. The spectrum collected at open circuit potential (OCP) in CO_2_-saturated 0.1 M KHCO_3_-D_2_O electrolyte was used as a background.

### Electrochemical measurements for CER

The experiments were performed in a custom-designed flow-cell system. The carbon paper electrode (the commercial Sigracet 29BC gas diffusion layers with the standard microporous layer based on 77% carbon black and 23% PTFE) was sprayed by airbrush with a loading catalyst of 1 mg/cm^2^. The geometric area of the electrode was set to 1 cm^2^. and Ni foam or IrO_2_/Ti mesh with IrO_2_ loading of 1 mg/cm^2^ (for studying the stability of cathode) were acted as working and counter electrode, respectively, which was separated by an anion exchange membrane (Fumasep, FAB-PK-130). The FAB-PK-130 is an anion exchange membrane with a thickness of 130 μm, which is cut to 1.5 cm * 1.5 cm size for practical use. The purchased FAB-PK-130 membrane was placed in 1 M KOH solution for 72 h to activate the anion exchange and subsequently used directly in electrochemical tests. The Hg/HgO electrode was used as a reference electrode. The relevant electrochemical tests were performed in 1 M KOH (or 0.1, 0.5, 1 M KHCO_3_, 2 M KOH, 3 M KCl) with using a Corrtest Workstation. During the measurements, CO_2_ was directly fed to the back of cathode GDE at a rate of 20 sccm. The electrolyte was forced to continuously circulate through the chamber at a rate of 10 sccm. All the applied cathode potentials after *iR* cell compensation were converted to the RHE reference scale using ${E}_{R{HE}}={E}_{{Ag}/{AgCl}}+0.204V+0.0591\times {pH}-0.85\times i\times R$ (*i*: applied current; *R*: cell resistance). The electrochemical impedance spectroscopy (EIS) measurement for measuring the ohmic loss between the working and reference electrodes was performed with frequency ranges from 100,000 to 0.1 Hz and an amplitude of 5 mV at open-circuit voltage in three-electrode system. The linear sweep voltammetry (LSV) curves were carried with a scan rate of 5 mV s^−1^. Controlled potential electrolysis was performed at each potential for 10 min.

### CER product analysis

The collected products were analyzed via gas chromatography (GC) and ^1^H NMR on a 400 MHz NMR spectrometer. The gaseous products of CO_2_ reduction, including carbon monoxide, methane, ethylene, ethane, and propylene, are detected and quantified using GC with an FID detector equipped with a nickel conversion furnace. Hydrogen is quantitatively detected using a TCD detector. The liquid products, including methanol, ethanol, n-propanol, formic acid, and acetic acid, are quantitatively detected using NMR with dimethyl sulfoxide serving as the internal standard. Typically, 0.5 mL KOH electrolyte after electrolysis was mixed with 100 μL of D_2_O and 67 μL of DMSO, including 5 mM as an internal standard. The ^1^H NMR spectrum was measured with water suppression via a pre-saturation method. The faradaic efficiency is calculated based on the calibration curve as follows:

1 $${N}_{{products}}={C}_{{products}}\times V\times {N}_{A}\times {ne}$$

2 $${N}_{{total}}=\frac{Q}{e}$$

3 $${FE}=\frac{{N}_{{products}}}{{N}_{{total}}}\times 100\%$$

*where* ${N}_{{products}}$ is total number of product transfer electron, ${C}_{{products}}$ is the concentration of product, *V* is the volume of electrolyte or gases, *N*_*A*_ : avogadro constant, 6.022 × 10^23^ mol^−1^, *n* is the number of electron transferred for product formation, *e* is electron, *Q* is the number of transfer charge, ${N}_{{total}}$ is total number of transfer electron.

The energy efficiency (EE) was defined as the ratio of fuel energy to applied energy, which was calculated for the half-cell of CRR with the following equation:

4 $$EE\left(\%\right)=\frac{{E}_{{products}}^{0}}{{E}_{{products}}^{{applied}}}\times {{FE}}_{{products}}\times 100\%$$

Where ${E}_{{ethanol}}^{0}$ = 0.07 V, ${E}_{n-{propanol}}^{0}$= 0.1 V is the thermodynamic potential of CRR to ethanol and n-propanol, ${{FE}}_{{products}}$ are the faradaic efficiencies of ethanol and n-propanol productions at an applied potential. ${E}_{{products}}^{{applied}}$ is the applied potential for alcohols production.

The production rate for formate was calculated using the following equation:

5 $${Yi}{eld\; rate}=\frac{Q\times {{FE}}_{{alcohols}}}{F\times n\times t\times S}$$

where *Q* is the total charge passed, *t* is the time (1 h) and *S* is the geometric area of the electrode (1 cm^2^).

The partial current densities (${j}_{a{lcohols}}$) of products were calculated as below, where *S* is the geometric area (1 cm^2^) of the cathode, *i* is the current of the electrode:

6 $${j}_{a{lcohols}}=\frac{i\times {{FE}}_{{alcohols}}}{S}$$

### Density functional theory (DFT) calculations

All calculations in this study were performed using the Vienna ab initio simulation package (VASP) based on density functional theory (DFT)^70^. We employed projector augmented wave (PAW) pseudopotentials and the Perdew–Burke–Ernzerhof (PBE) exchange-correlation functional within the semi-local generalized gradient approximation (GGA)^71^. To adequately capture weak long-range van der Waals (vdW) interactions, we employed an empirical dispersion-corrected DFT method (DFT-D3)^72^. The kinetic energy cutoff for the plane wave expansion was set to 500 eV. The self-consistent field (SCF) iteration was considered to converge when the threshold reached 10^−5^ eV. Geometry optimization was performed using the conjugate gradient method, with forces on each atom constrained below 0.03 eV Å^−1^.

The Cu/Cu_2_O model was obtained by selectively removing specific oxygen atoms from the Cu_2_O(111) surface unit cell, adopting a p(4*2) Cu_2_O(111) unit cell. Subsequently, we optimized the lattice parameters and atomic positions. The optimized Cu/Cu_2_O composite exhibited lattice parameters of *a* = 21.48 Å, *b* = 10.29 Å, *c* = 23.62 Å, *α* = 90°, *β* = 90°, *γ* = 118.63°. To incorporate chlorine (Cl), an O atom on the surface was replaced with a Cl atom, yielding the Cl-Cu/Cu_2_O model. For the Cu/Cu_2_O composite slab models, we employed a 1 × 2 × 1 k-point mesh. The Cu_2_O(111) model was based on a p(2*2) unit cell, while the Cu(111) model also utilized a p(2*2) unit cell. The atomic coordinates of the optimized computational models are provided in Supplementary Dataset 1, which are defined as Cu-POSCAR, Cu_2_O-POSCAR, Cu_P_/Cu_2_O-POSCAR, Cu_L_/Cu_2_O-POSCAR. To take into account the on-site Coulomb interaction between 3d electrons of Cu, the GGA+U approach was also employed with a U–J value of 4 eV^73,74^. Note that Cu(111) does not use GGA+U. The reaction free energy change and adsorption energy can be obtained with the equation below:

7 $$\Delta G=\Delta E+\Delta {ZPE}-T\Delta S$$

8 $$\Delta {E}_{a{ds}}={E}_{*X}-{E}_{*}-{E}_{X}$$

Where $\Delta E$ represents the total energy difference before and after the intermediate is adsorbed, $\Delta {ZPE}$ and $\Delta S$ denote the differences in zero-point energy and entropy, respectively. The zero-point energy and entropy of the free molecules and adsorbents were derived from vibrational frequency calculations. ${E}_{*X}$ corresponds to the total energy of the system when molecule *X* is adsorbed on the surface of the slab, ${E}_{*}$ represents the energy of the slab system, and ${E}_{X}$ signifies the energy of the adsorbed intermediate *X*.

### Supplementary information

Supplementary Information

Peer Review File

Description of Additional Supplementary Files

Supplementary Data 1

### Source data

Source Data

## Data Availability

The raw data of the figures in the main manuscript are available in figshare with the identifier(s) 10.6084/m9.figshare.25124129. All other data needed to evaluate the conclusions in the paper are present in the paper and the Supplementary Information or can be obtained from the corresponding authors upon request. All data are available in the manuscript, the supplementary materials, and from the authors on request. Source data are provided with this paper.

## Code availability

The code used in this work can be obtained from the corresponding authors on request.
