# Supplementary material for: Low-coordinated copper facilitates the *CH2CO affinity at enhanced rectifying interface of Cu/Cu2O for efficient CO2-to-multicarbon alcohols conversion
Source: Nat Commun. 2024 Jun 18;15:5172. doi: 10.1038/s41467-024-49247-4 (PMC11189494; doi:10.1038/s41467-024-49247-4)
Supplement: Supplementary file 1 — Supplementary Information [file 41467_2024_49247_MOESM1_ESM.pdf]

Supplementary Materials

Low-coordinated copper facilitates the *CH_2CO affinity at enhanced rectifying interface of Cu/Cu_2O for efficient CO_2-to-multicarbon alcohols conversion

Yangyang Zhang,[1,2] Yanxu Chen,[1,2] Xiaowen Wang,[1] Yafei Feng,[1] Zechuan Dai,[1] Mingyu Cheng[1] and Genqiang Zhang*[1]

Affiliations

[1]Hefei National Research Center for Physical Sciences at the Microscale, CAS Key Laboratory of Materials for Energy Conversion, Department of Materials Science and Engineering, University of Science and Technology of China, Hefei, Anhui 230026, China.

Email: gqzhangmse@ustc.edu.cn

[2]These three authors contribute equally to this work.

Supplementary Figures:

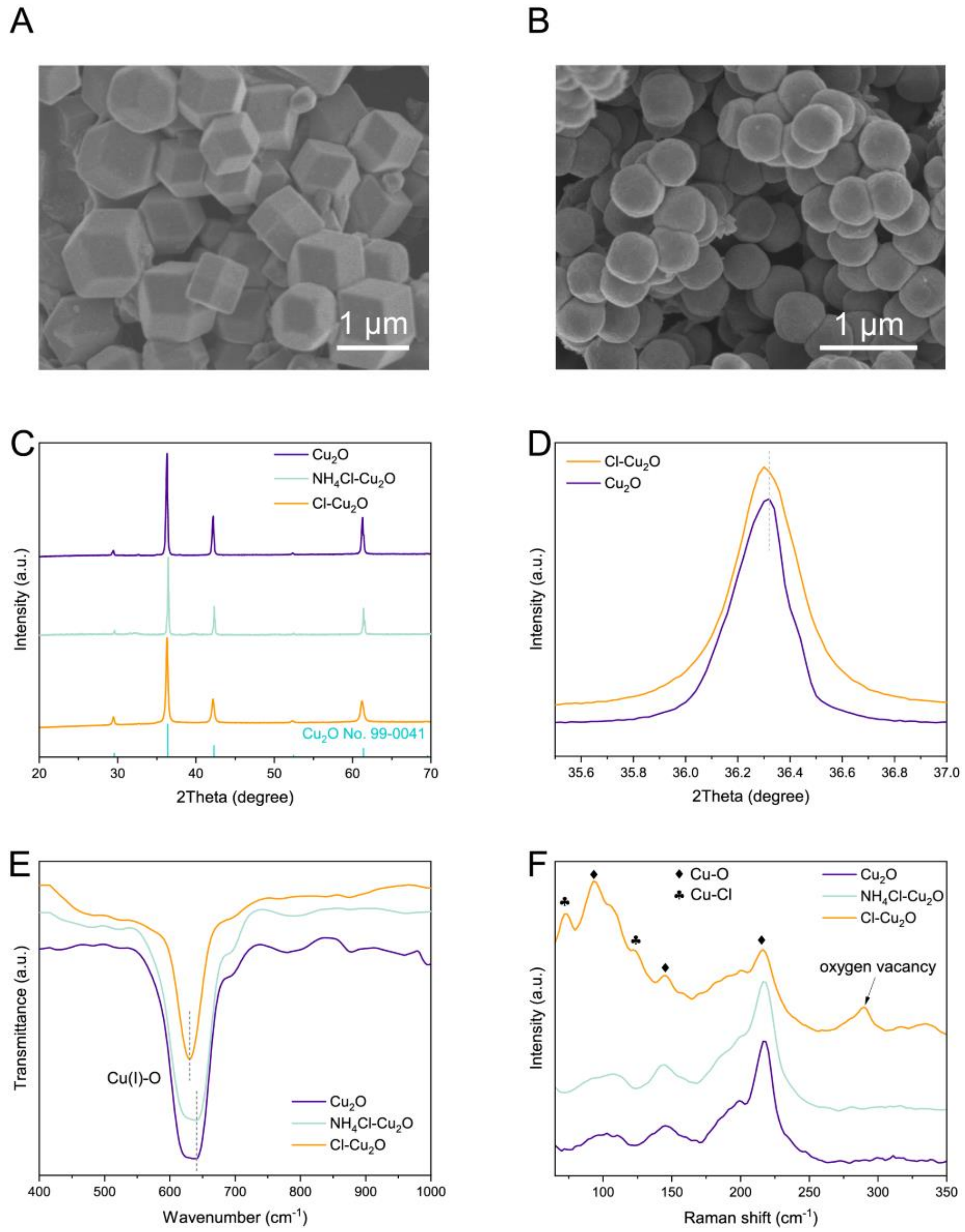

Supplementary Figure 1 | The morphology and structure of Cl-Cu_2O. (A and B) SEM images of as-synthesized Cu_2O (A) and Cl-Cu_2O (B). (C and D) XRD patterns of precursors (C) and cooresponding enlarge zoom-in patterns of (111) peak in Cu_2O and Cl-Cu_2O (D). (E) FT-IR and (F) Raman patterns of precorsors.

Typically, hydroxylamine hydrochloride and glucose were used as reducing agents with or without chlorine source for preparing Cl-Cu_2O and Cu_2O (Supplementary Fig. 1 and 2). Compared to Cu_2O powders, the Cl-Cu_2O nanoparticles show smaller crystallite size (400nm, a= 4.2756 Å, b= 4.2756 Å and c= 4.2756 Å) and homogeneous distribution of Cl element (Supplementary Fig. 3). Moreover, the content of Cl atom keeps a stable value with a deeper Ar^+ etching (Supplementary Fig. 4 and Supplementary Table 1). obviously, the broadened

diffraction peaks of Cl-Cu_2O are shifted to a lower 2θ angle, which reflects a slight lattice expansion and smaller crystallite size resulted from doping Cl atoms. Moreover, the very strong band at 630 cm^{-1} of Fourier-transform infrared (FT-IR) spectroscopy is attributed to the stretching vibration of Cu(I)–O of Cl-Cu_2O, which appears a slight red shift due to the restricted vibration frequency.[1] The relevant Raman experiment also proves the doping Cl atoms deeply inside Cl-Cu_2O instead of simply adsorbing on the surface of Cu_2O, such as NH_4Cl-Cu_2O, where the signals of Cu−Cl bond (71 and 122 cm^{-1}) only exist in Cl-Cu_2O.

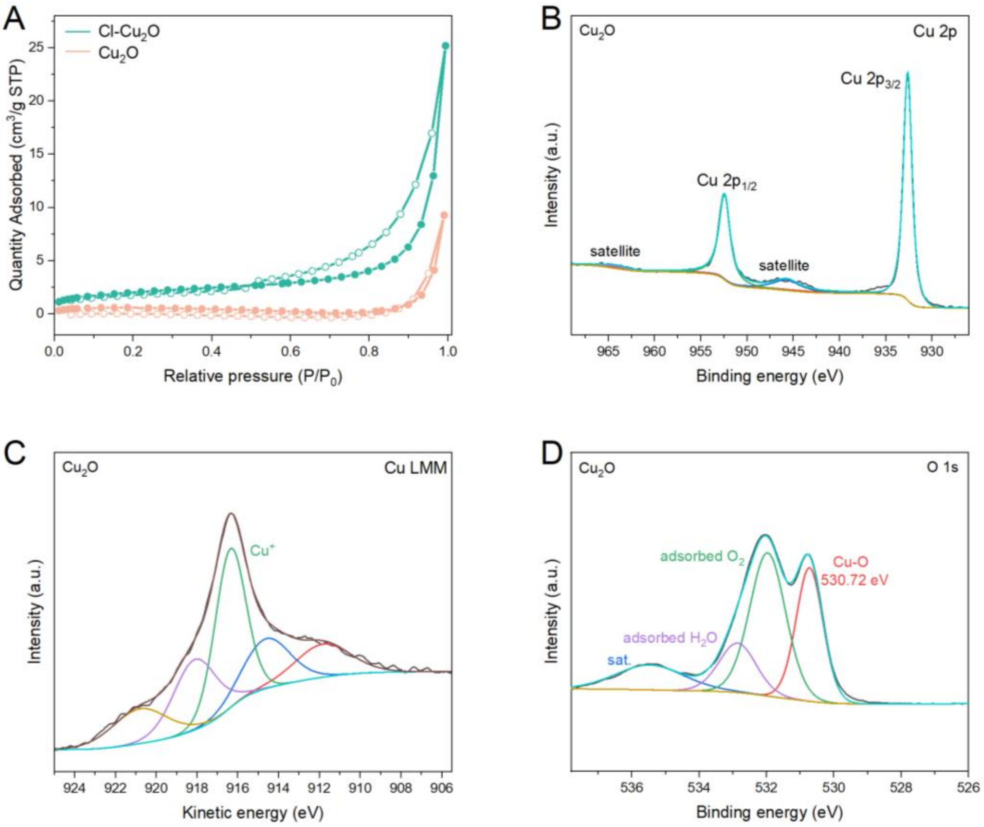

Supplementary Figure 2 | Nitrogen absorption and adsorption curves and High-resolution XPS. (A) Nitrogen absorption and adsorption curves of as-synthesized Cu_2O and Cl-Cu_2O, (B) High-resolution Cu 2p, (C) Cu LMM and (D) O 1s of as-synthesized Cu_2O.

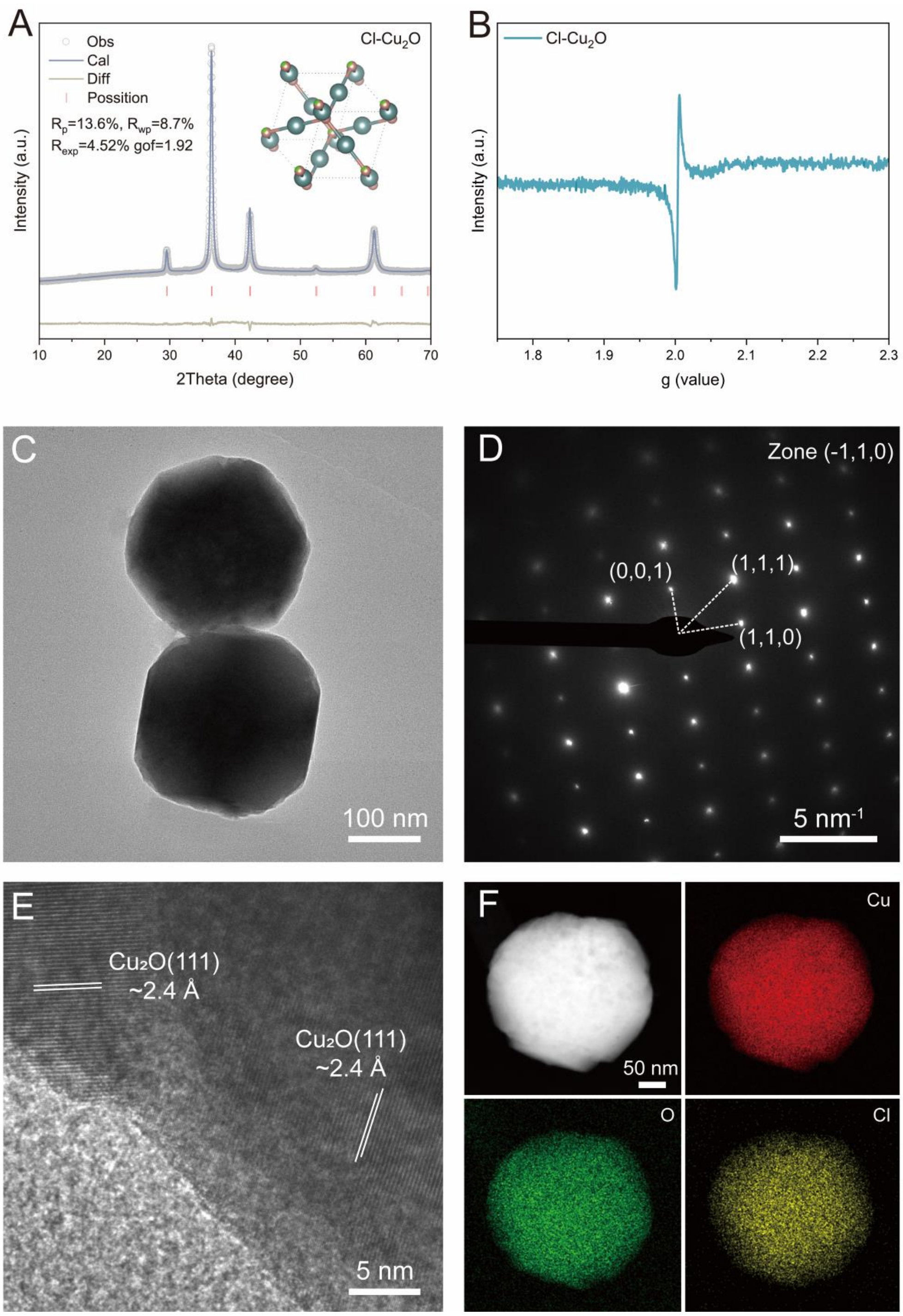

Supplementary Figure 3 | The refined structure of Cl-Cu_2O. (A) Powder XRD profiles and Rietveld refinement patterns and corresponding bat model of Cl-Cu_2O. (B) EPR spectra of Cl-Cu_2O. (C) TEM image, (D) SAED, (E) HRTEM analysis and (F) corresponding elemental mapping results.

The Rietveld refinement indicates that the lattice parameters of Cl-Cu_2O show a slightly expansive volume compared to that of Cu_2O (a= 4.269 Å, b= 4.269 Å and c= 4.269 Å), originating from the increased Cl^--Cu^+-O^{2-} electrostatic repulsions in the lattice cell. The theoretical content of Cl (~4 at%) is basically consistent with the experimental value of 3.64 at% (Table S2). The EPR spectra of Cl-Cu_2O reveals the deficiencies of oxygen atoms due to

the introduce of Cl. The transmission electron microscopy (TEM) images reveal the exposure of monocrystalline Cu_2O (111) facets and uniform distribution of Cl.

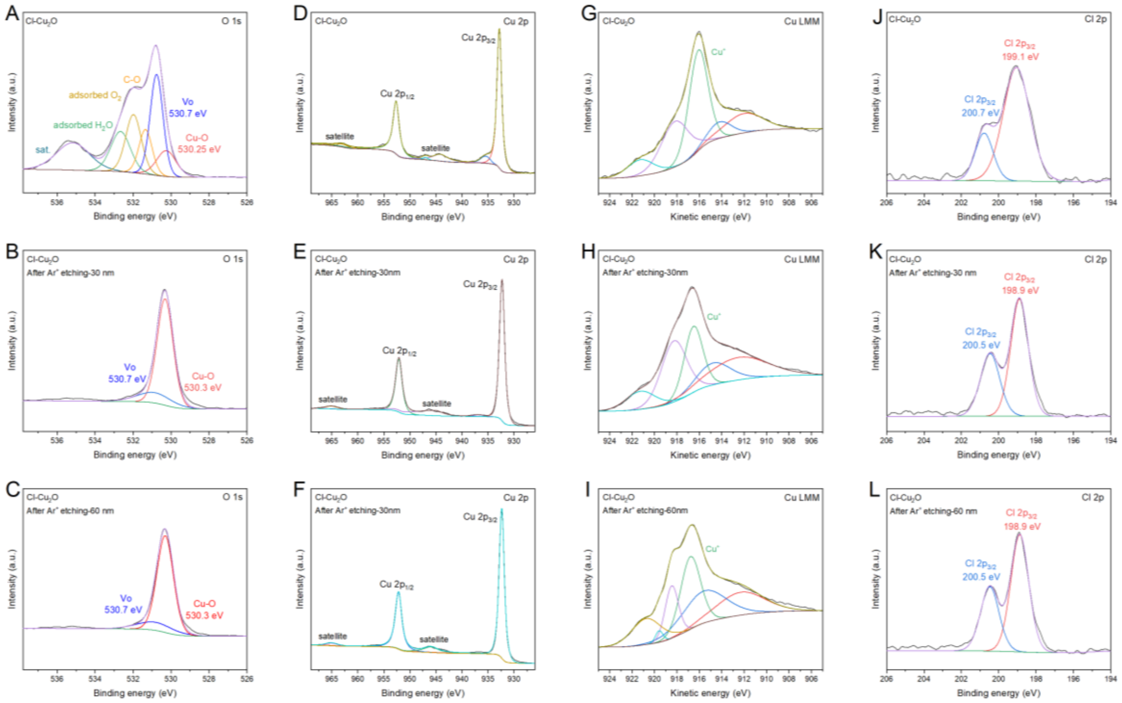

Supplementary Figure 4 | The XPS spectra of Cl-Cu_2O. (A-C) High-resolution O 1s, (D-F) Cu 2p, (G-I) Cu LMM and (J-L) Cl 2p XPS patterns of Cl-Cu_2O with or without Ar^+ etching.

X-ray photoelectron spectroscopy (XPS) was carried to estimate the electron structure of Cl-Cu_2O. The peak intensity of Cl keep a stable level after Ar^+ etching, which implies that some Cu-O bonds are substituted by Cu-Cl and leave a little oxygen vacancies.

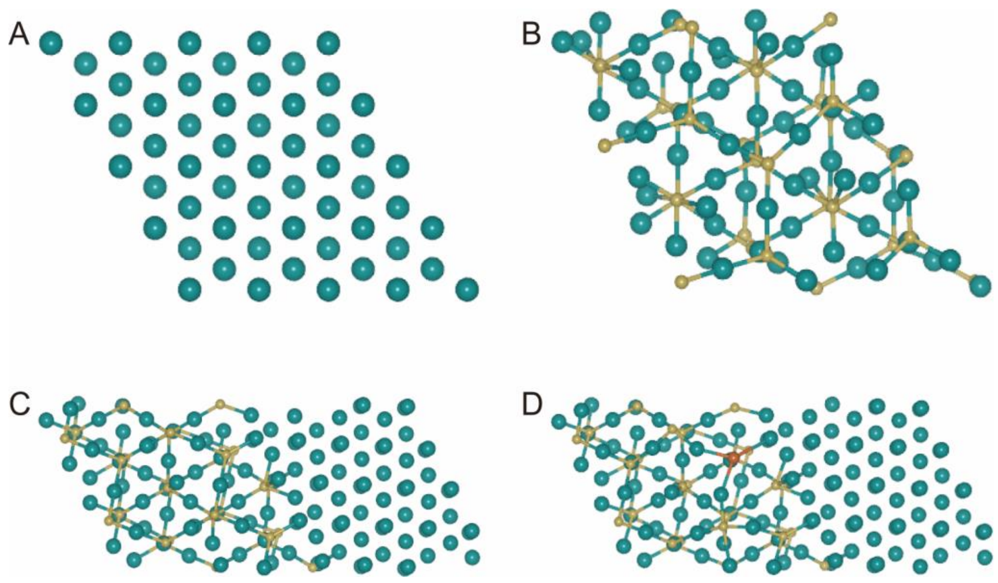

Supplementary Figure 5 | The simulated slab models. (A) Cu, (B) Cu_2O, (C) Cu_P/Cu_2O and (D) Cu_L/Cu_2O.

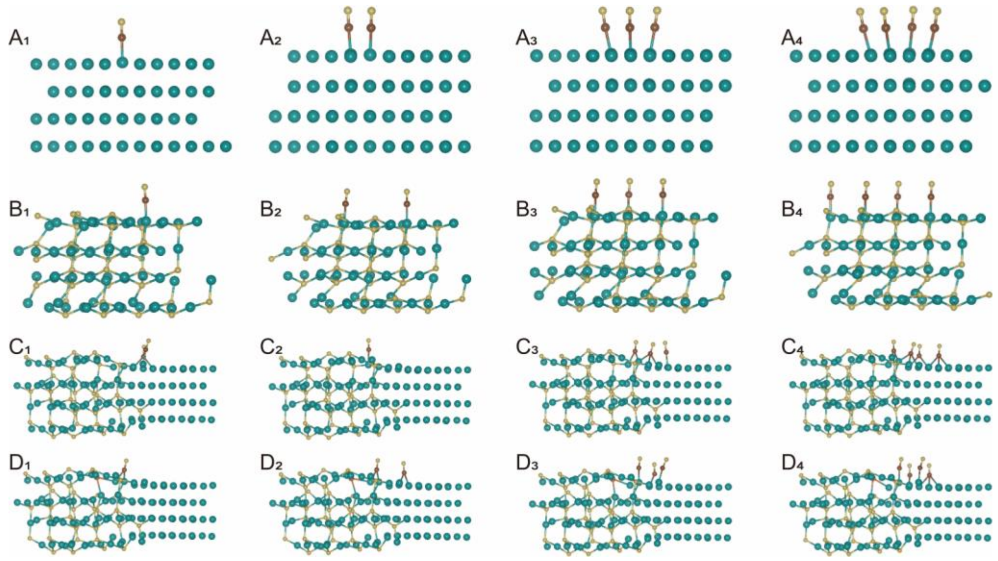

Supplementary Figure 6 | The adsorption models of various *CO intermidates. (A_1-A_4) Cu, (B_1-B_4) Cu_2O, (C_1-C_4) Cu_P/Cu_2O and (D_1-D_4) Cu_L/Cu_2O.

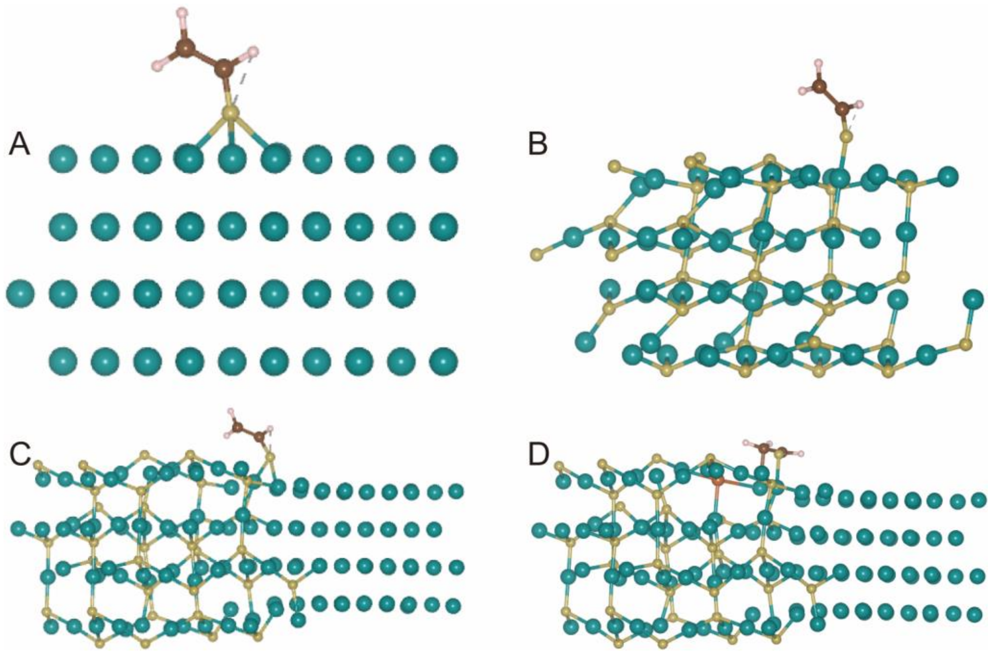

Supplementary Figure 7 | The adsorption models of *CH_2CHO intermidate. (A) Cu, (B) Cu_2O, (C) Cu_P/Cu_2O and Cu_L/Cu_2O (D).

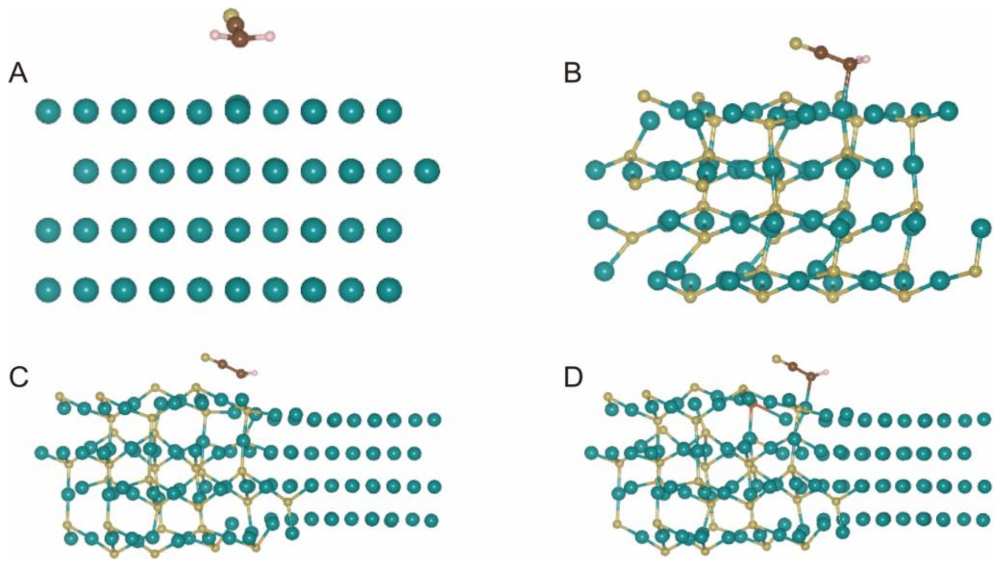

Supplementary Figure 8 | The adsorption models of *CH_2CO intermidate. (A) Cu, (B) Cu_2O, (C) Cu_P/Cu_2O and Cu_L/Cu_2O (D).

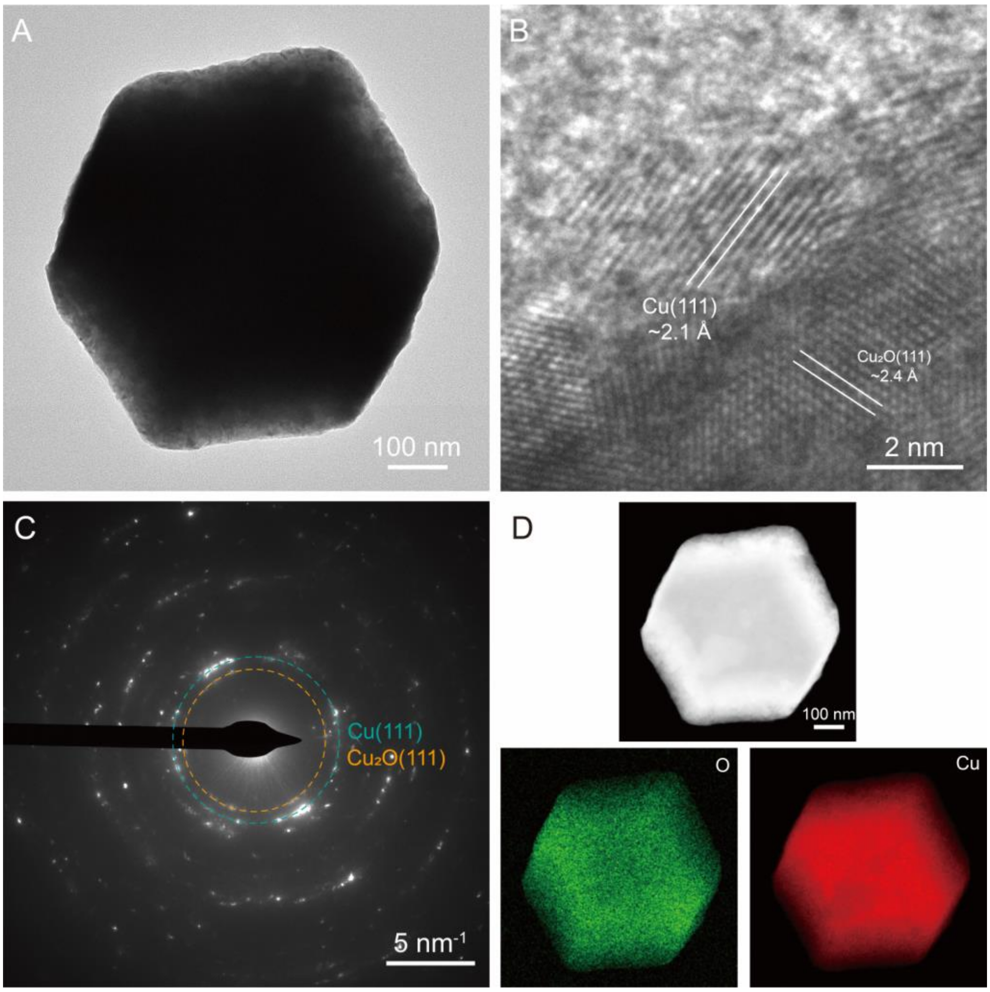

Supplementary Figure 9 | (A) TEM image, (B) HRTEM analysis, (C) SAED and (D) corresponding elemental mapping results of Cu_P/Cu_2O.

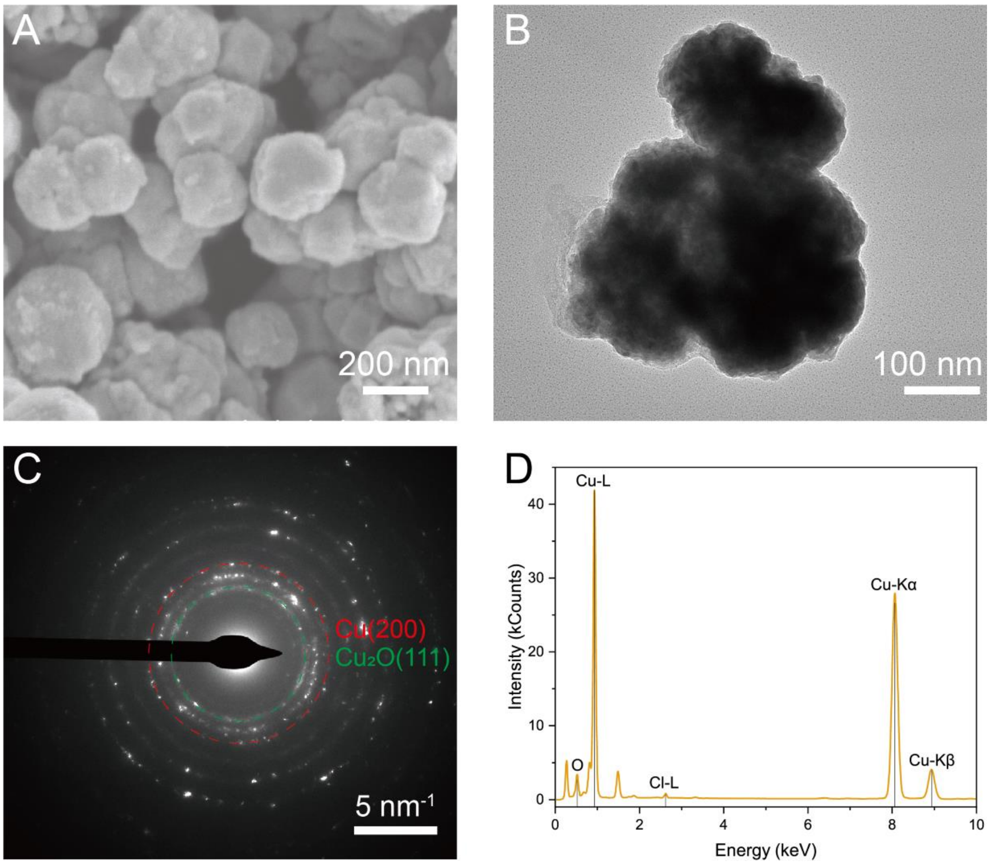

Supplementary Figure 10 | (A) SEM, (B) TEM, (C) SAED and (D) the extracted EDS spectrum of Cu_L/Cu_2O.

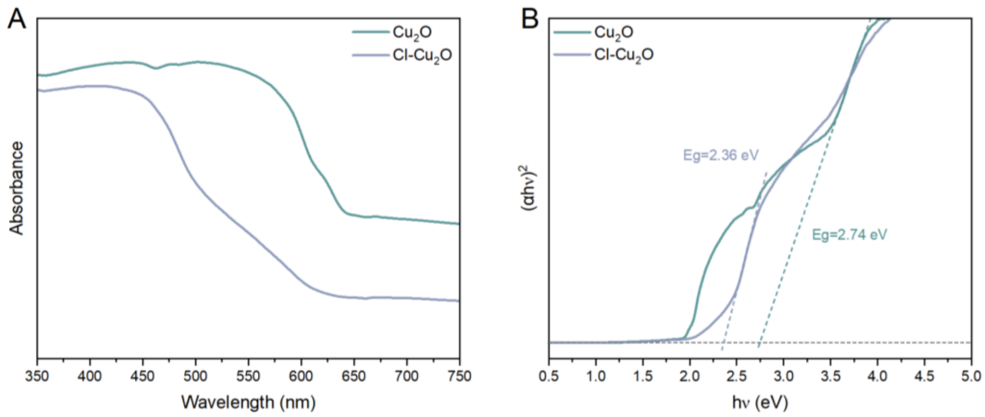

Supplementary Figure 11 | (A) UV−vis diffuse absorption spectrum of Cu_2O and Cl-Cu_2O and (B) cooresponding Tauc plots $(\alpha\hbar v)^2$ versus light energy ($\hbar$v) derived by transforming the Kubelka−Munk function.

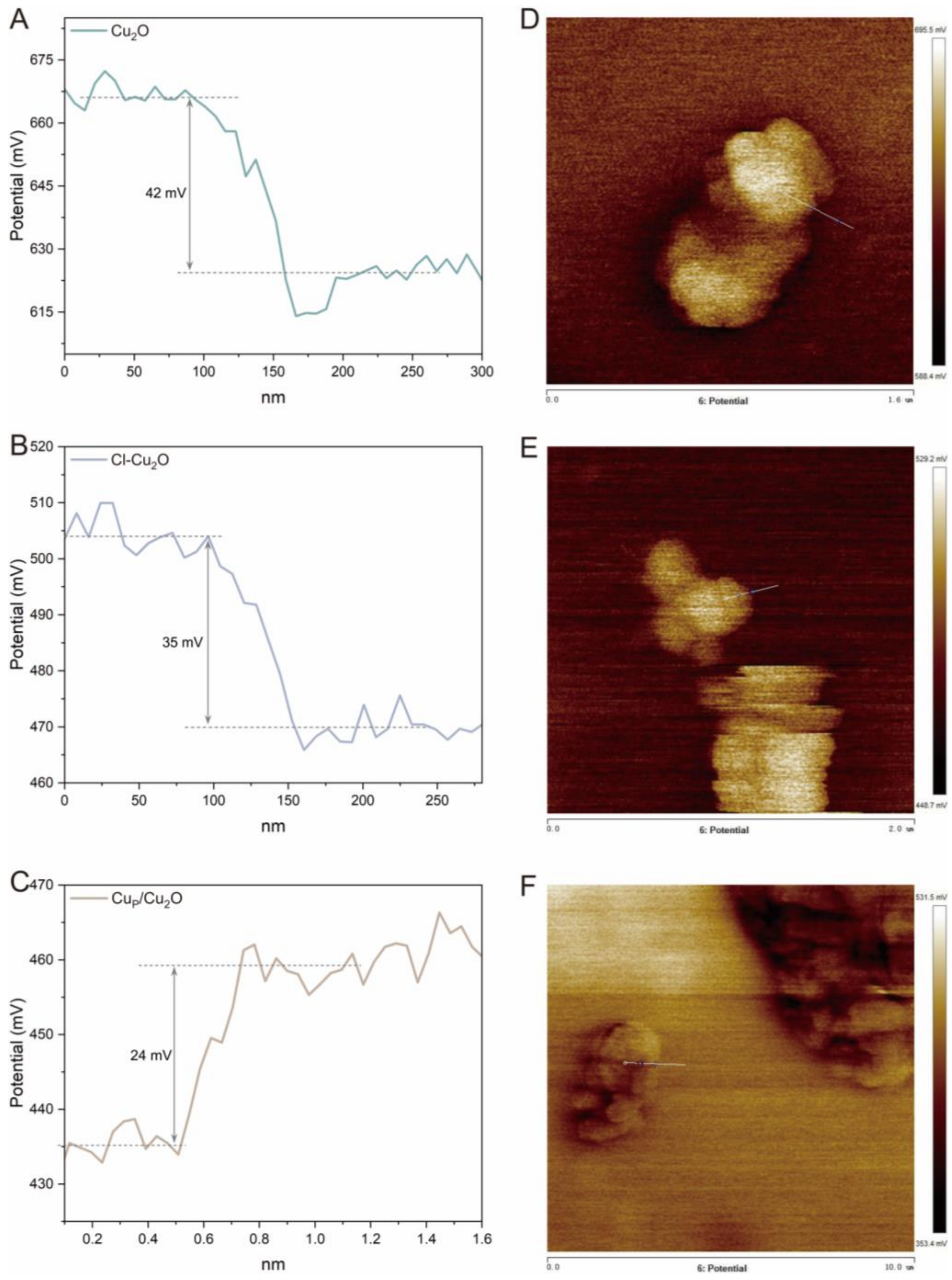

Supplementary Figure 12 | The surface potential and corresponding height profiles and potentials along the white line accordingly for Cu_2O (A, B), Cl-Cu_2O (C, D), Cu_P/Cu_2O (E, F).

The topography and surface potential map of samples were characterized simultaneous by using KPFM.[2] The topographies and surface potentials were measured by KPFM based on dynamic force microscopy principles to give work functions of the samples. The work function ϕ_{sample} was further calculated from the surface potential according to equation:

$$\phi_{Sample} = \phi_{Tip} + qV_{CPD} \quad (1)$$

$$V_{CPD} = V_{CPD\text{-}sample} - V_{CPD\text{-}} \quad (2)$$

$$\phi_{Sample} = = \phi_{substrate} + qV_{CPD} \quad (3)$$

where $V_{CPD\text{-}sample}$, $V_{CPD\text{-}substrate}$ are the contact potential difference of sample and substrate measured by KPFM in volts. ϕ_{Tip}, ϕ_{sample} and $\phi_{substrate}$ are the work functions of tip, sample and substrate in eV, respectively and q is the electronic charge. The work functions of tip can be calculated by measuring gold samples by KPFM.

The substrate is SiO_2 (WF=5.05 eV) and the standard gold disk with a work function of 5.3 eV is used to calibrate the probe. Figure 2E and Supplementary Figure 12 exhibite the contact potential difference (CPD) profiles for each material, where the average surface potential can be estimated to be 0.042, 0.035, 0.024 and 0.037 V for Cu_2O, Cl-Cu_2O, Cu_P/Cu_2O and Cu_L/Cu_2O, respectively. From the CPD profiles, the work function of Cu_2O, Cl-Cu_2O, Cu_P/Cu_2O and Cu_L/Cu_2O samples are determined to be 5.1, 5.08, 5.03 and 5.01eV, respectively. These values are in accordance with the trends of work function calculated by UPS.

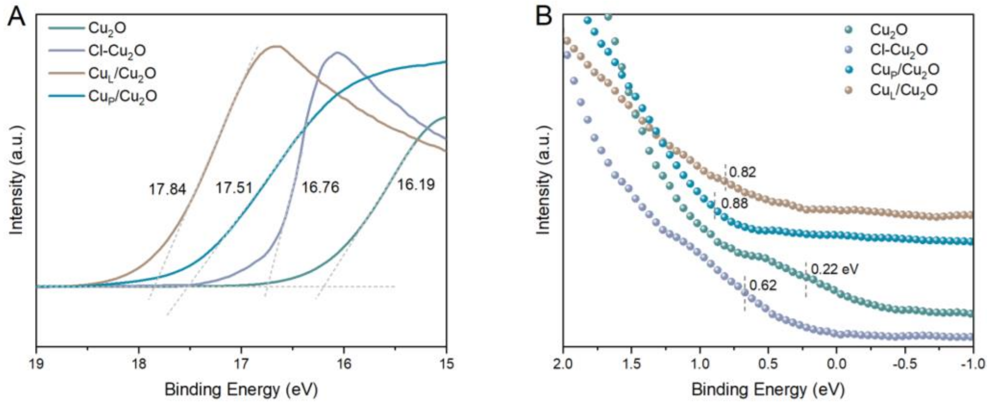

Supplementary Figure 13 | (A) Photoemission cutoff spectra and (B) Fermi edge of Cu_2O, Cl-Cu_2O, Cu_P/Cu_2O and Cu_L/Cu_2O.

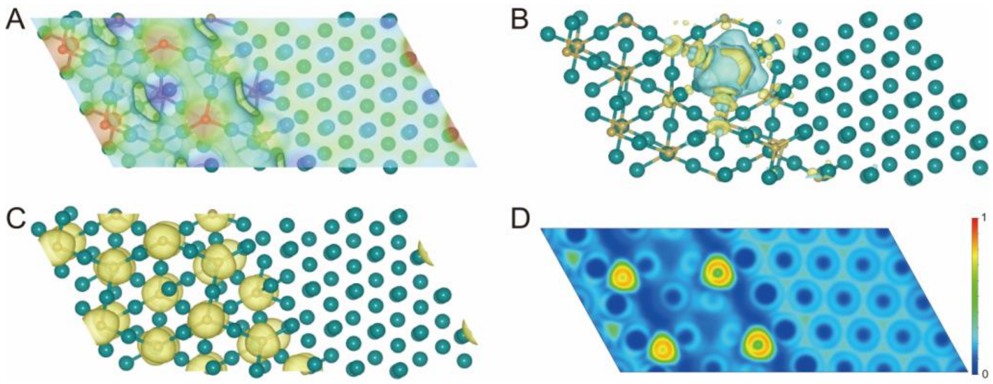

Supplementary Figure 14 | (A) electronstatic potential, (B) 3D electron density difference distributions, (C) electron localization function and (D) cooresponding 2D date display of Cu_P/Cu_2O.

The electron clouds around the side of Cu^+ at the rectifying interface of Cu_P/Cu_2O are decentralized owning to the weak lattice shrinkage and perfect lattice array, indicating the poor adsorption for intermediates.

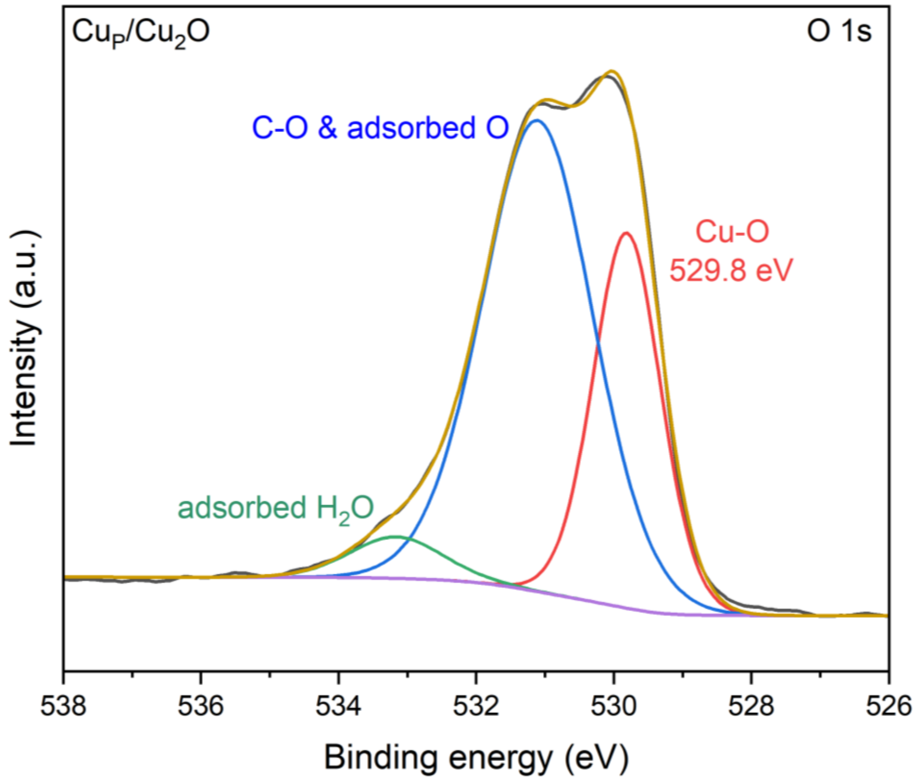

Supplementary Figure 15 | High-resolution O 1s XPS pattern of Cu_P/Cu_2O.

The O 1s XPS spectra is deconvoluted into three peaks situated at 529.8, 531.1 and 533.2 eV corresponding to the lattice oxygen (Cu-O), adsorbed oxygen/C-O and adsorbed H_2O on Cu_P/Cu_2O samples, respectively.

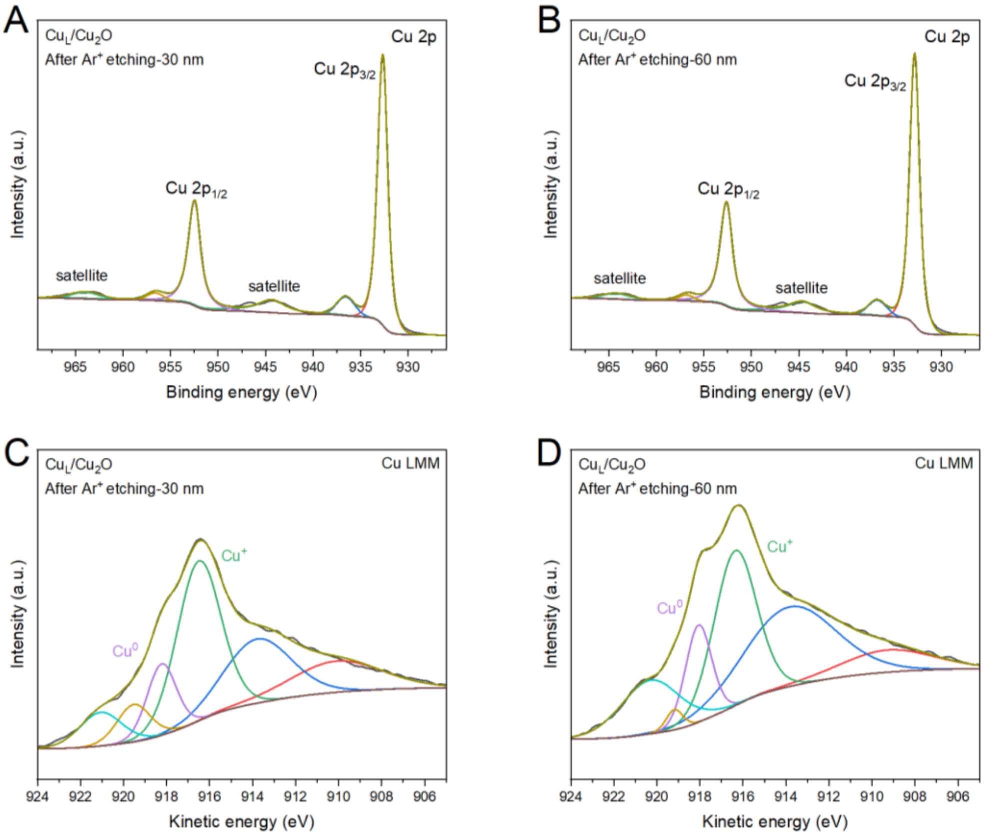

Supplementary Figure 16 | (A and B) High-resolution Cu 2p and (C and D) Cu LMM XPS patterns of Cu_L/Cu_2O after Ar^+ etching.

The higher bonding energy of Cu may result from the electron-rich Cu^+ in the depth of Cu_L/Cu_2O nanoparticles. The residual Cl and O atoms of Cu_L/Cu_2O make a difference with that the strongly electronegative elements always accompany with oxygen-derived Cu and inhibit metallization of electron-rich Cu^+.

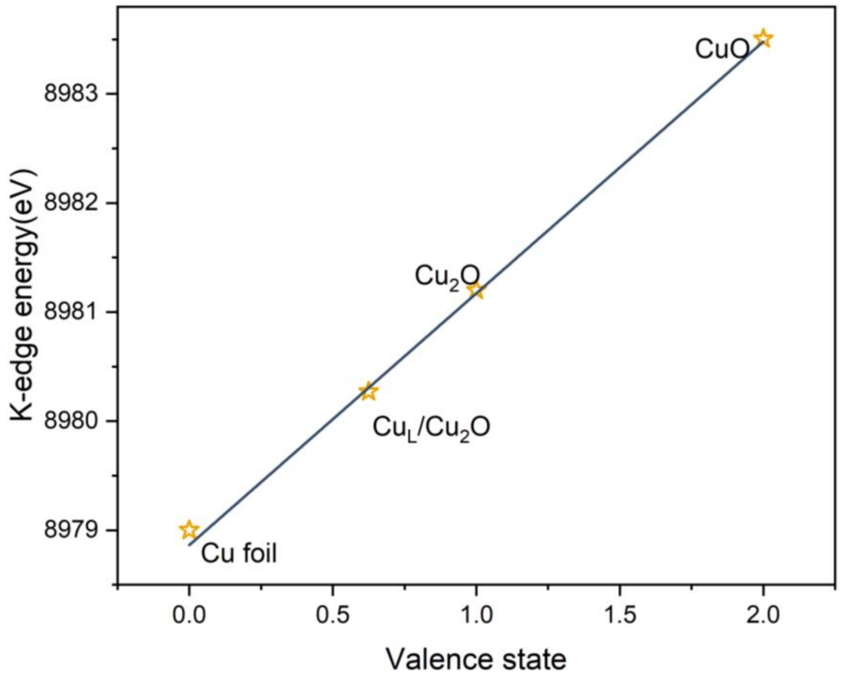

Supplementary Figure 17 | Chemical valence of Cu atom in Cu foil, Cu_2O, CuO and Cu_L/Cu_2O.

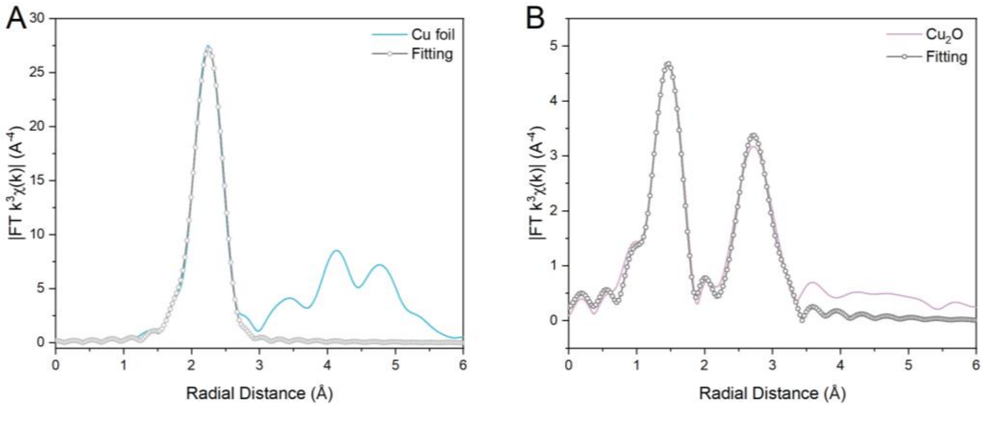

Supplementary Figure 18 | The fitting curves of k^3-weignted EXAFS spectra of pure Cu foil (A) and Cu_2O (B).

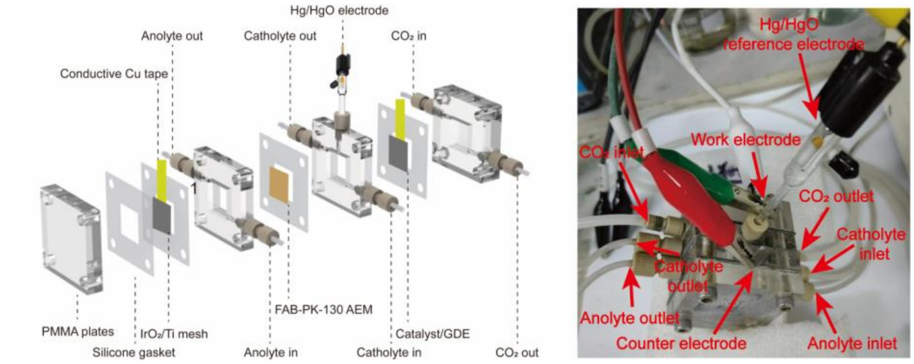

Supplementary Figure 19 | The three-electrode flow cell: schematic diagram (left) and optical image (right). GDL: gas diffusion layers. PMMA: polymethylmethacrylate.

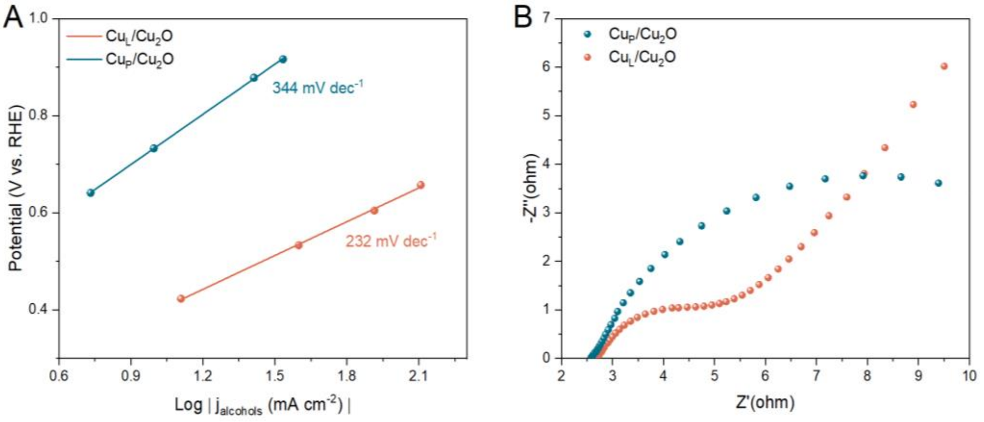

Supplementary Figure 20 | (A) Tafel curves and (B) EIS spectra of Cu_P/Cu_2O and Cu_L/Cu_2O, the system resistance has been tested three times.

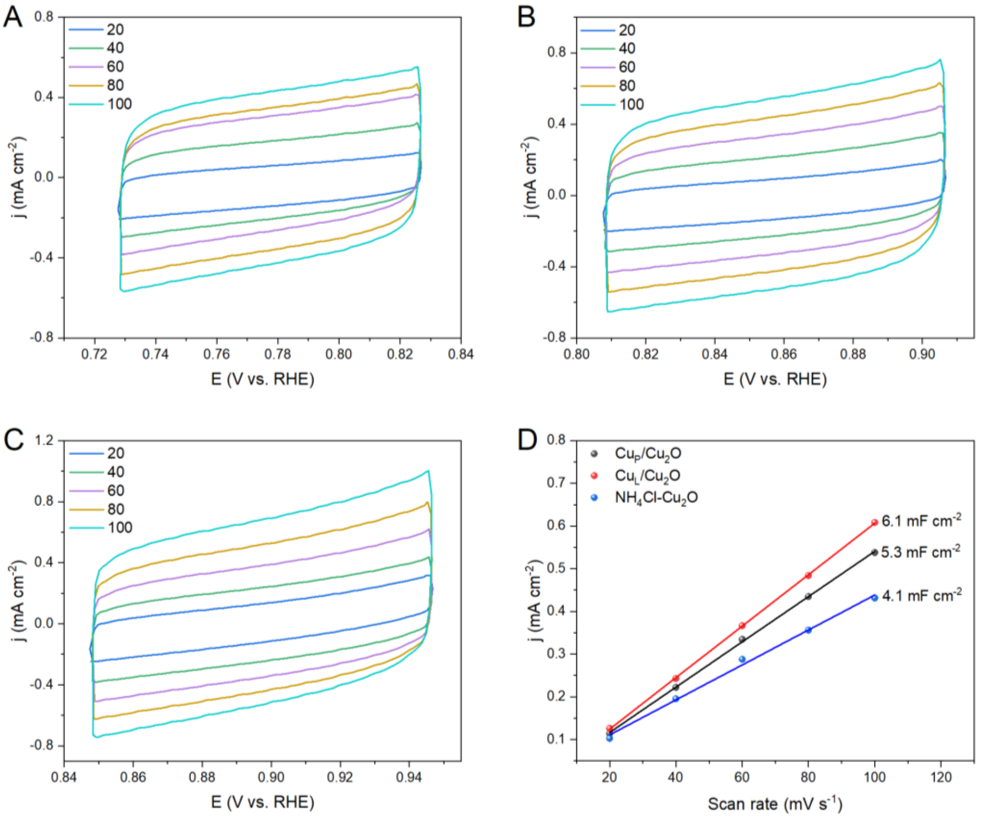

Supplementary Figure 21 | Electrochemical surface area (ECSA) measurement without iR compensation. Cyclic voltammagrams with various scan rates for NH_4Cl-Cu_2O (A), Cu_P/Cu_2O (B) and Cu_L/Cu_2O (C). (D) Current due to double-layer charging plotted against cyclic voltammetry scan rate.

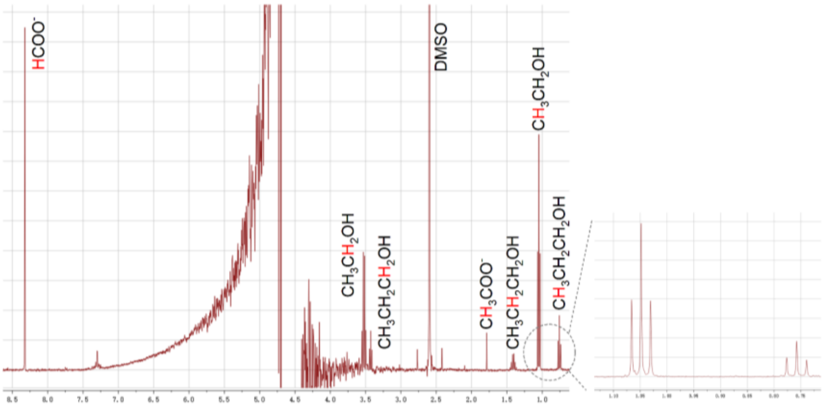

Supplementary Figure 22 | Representative 1 H NMR spectra of formate, acetate, ethanol and 1-propanol products using the Cu_L/Cu_2O catalyst at –200 mA·cm^{-2}.

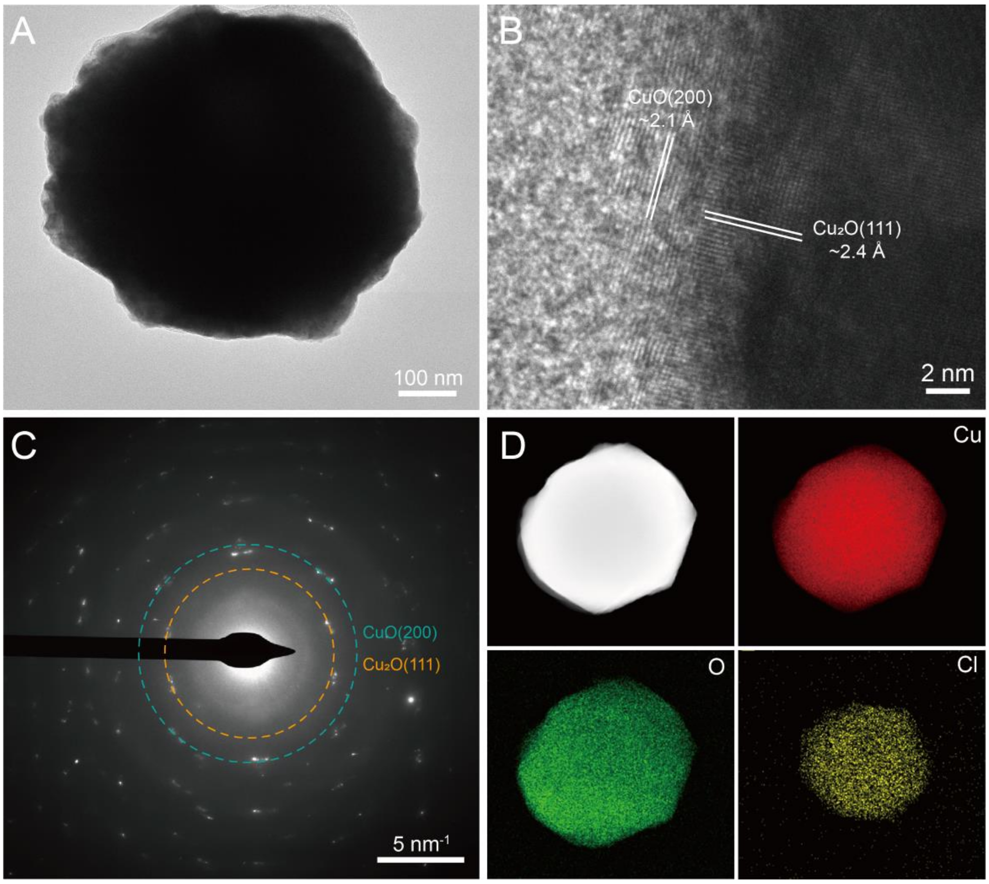

Supplementary Figure 23 | (A) TEM image, (B) HR-TEM analysis, (C) SAED and (D) corresponding elemental mapping results of NH_4Cl-Cu_2O.

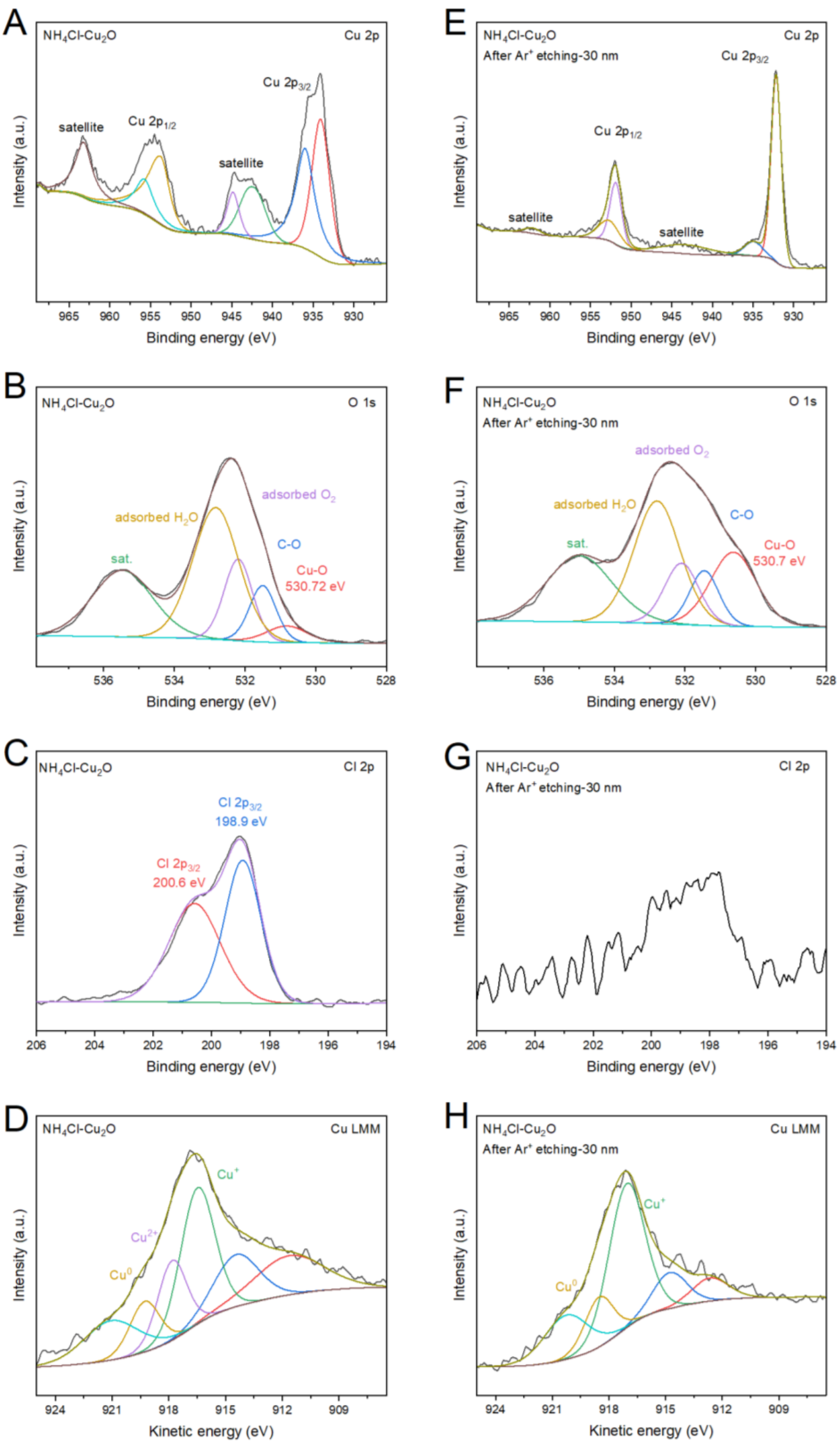

Supplementary Figure 24 | (A-E) Cu 2p, (B, F) O 1s, (C, G) Cl 2p and (D, H) Cu LMM XPS spectras of as-synthesized NH_4Cl-Cu_2O with or without Ar^+ etching.

The NH_4Cl-Cu_2O were oxidized during the immersing process, resulting in many Cu^{2+} on the surface of crystal. Ater Ar^+ etching, the Cl atoms only adhered on the surface of NH_4Cl-Cu_2O are exfoliated, which implies the physical adsorption of Cl element.

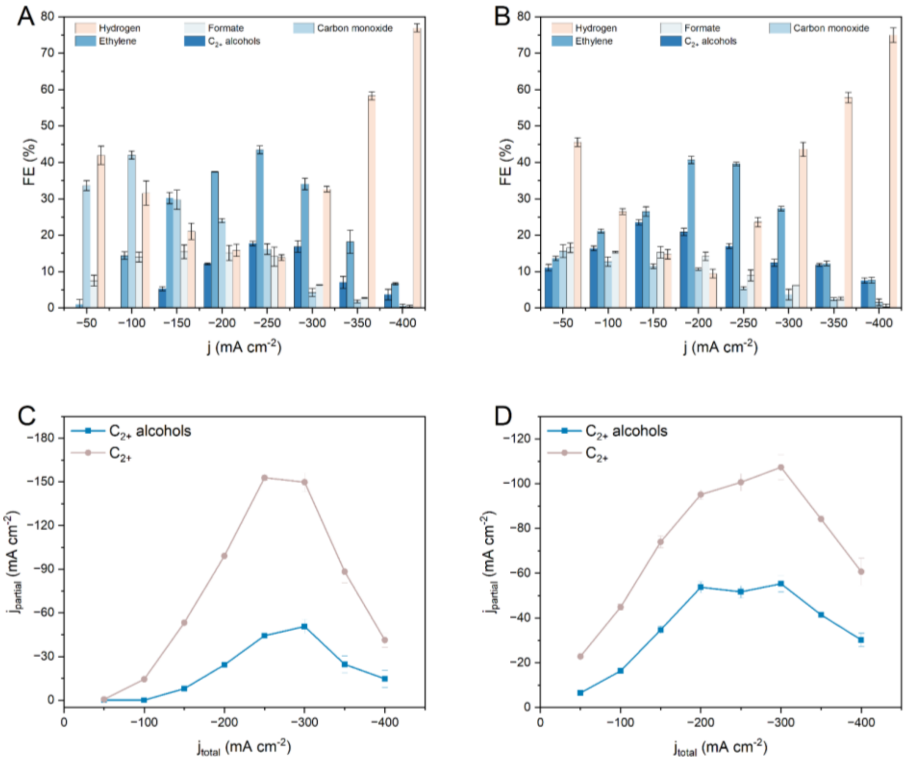

Supplementary Figure 25 | (A) FE and (C) partial current density of NH_4Cl-Cu_2O in 1 M KOH electrolyte. (B) FE and (D) partial current density of Cu_P/Cu_2O in 1 M KOH electrolyte with 3 M KCl. Error bars show the standard deviations calculated from three independent experiments.

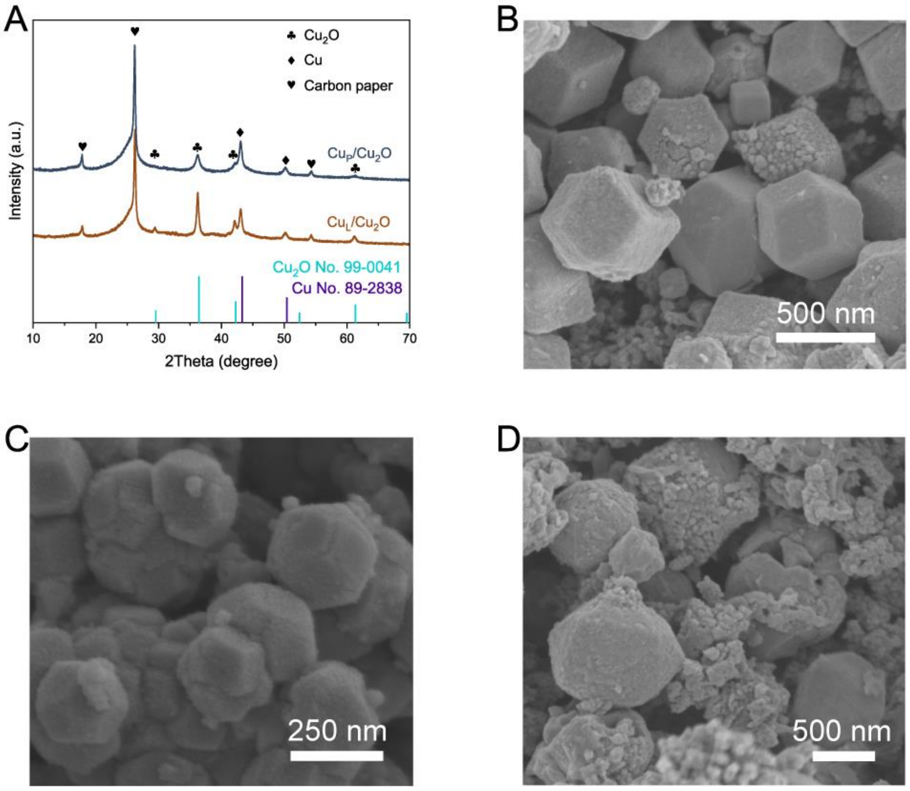

Supplementary Figure 26 | (A) XRD patterns of Cu_P/Cu_2O and Cu_L/Cu_2O catalysts after CER. SEM images of Cu_P/Cu_2O (B), Cu_L/Cu_2O (C) and NH_4Cl-Cu_2O (D) after CER.

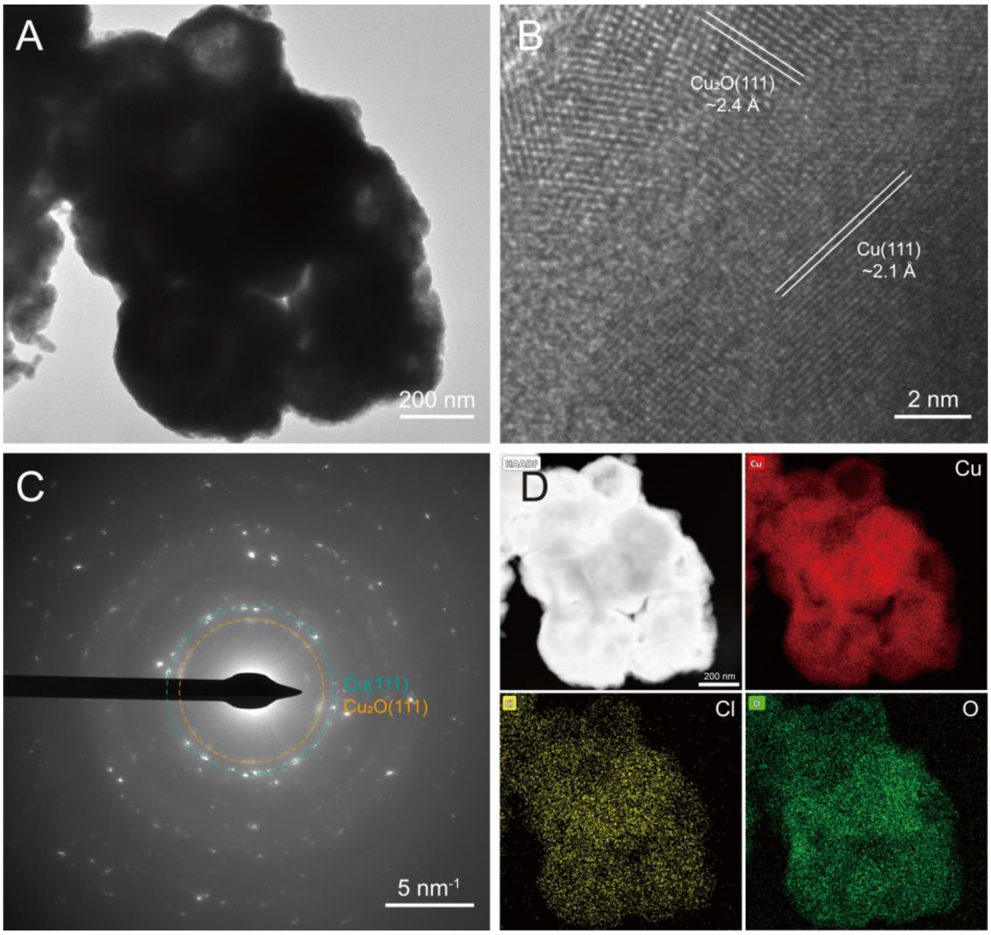

Supplementary Figure 27 | (A) TEM image, (B) HRTEM analysis, (C) SAED and (D) corresponding elemental mapping results of Cu_L/Cu_2O after CER.

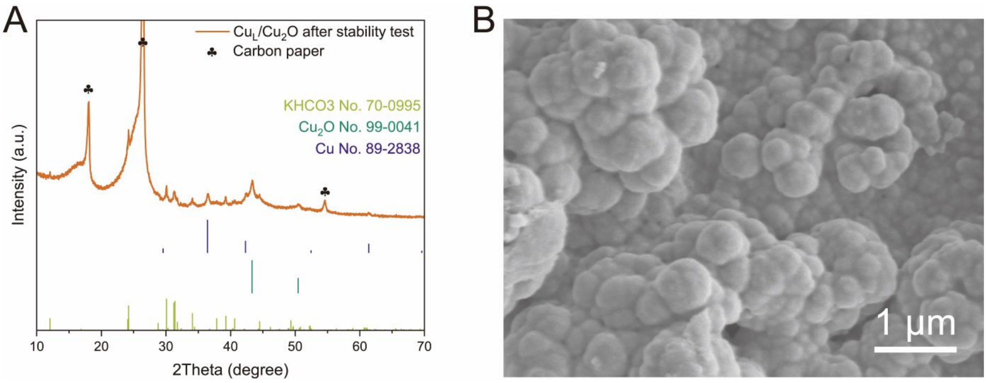

Supplementary Figure 28 | (A) XRD pattern and (B) SEM image of Cu_L/Cu_2O after long-term stability test of CER.

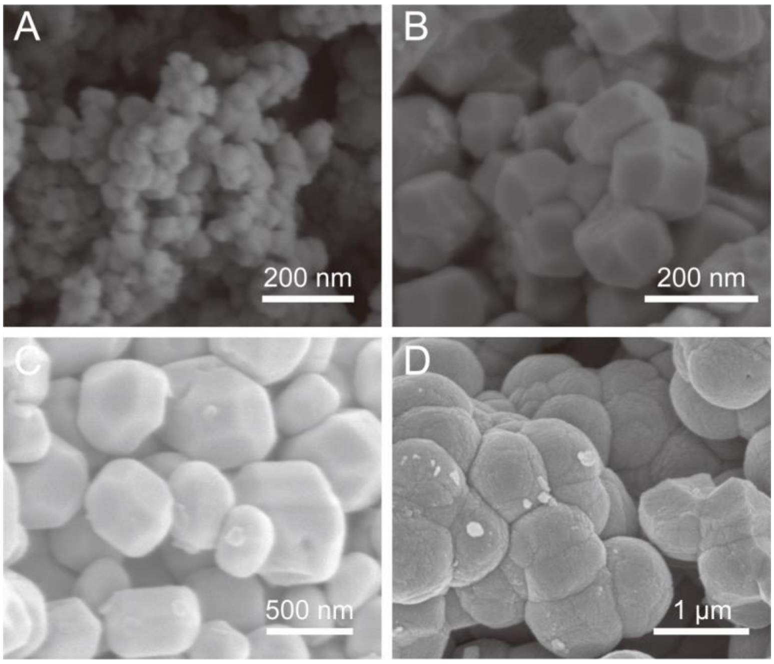

Supplementary Figure 29 | SEM images of Cl-Cu_2O with different particle size: (A) ~30 nm, (B) ~150 nm, (C) ~500 nm, (D) ~1μm

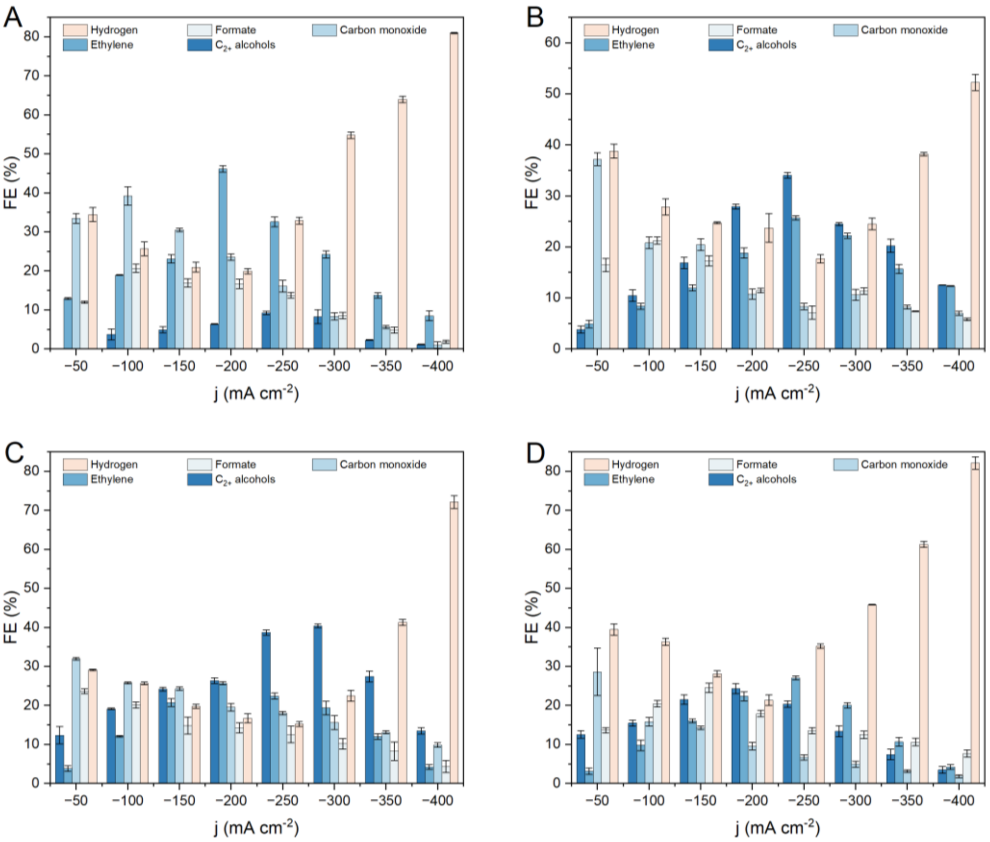

Supplementary Figure 30 | FE of products derived from Cl-Cu_2O with different particle size: (A) ~30 nm, (B) ~150 nm, (C) ~500 nm, (D) ~1μm in 1 M KOH electrolyte. Error bars show the standard deviations calculated from three independent experiments.

The Cl-Cu_2O powders with different particle sizes were prepared by modulating the inputs of reactant and reaction time. The morphological structure and electrochemical properties of samples were records in Supplementary Figure 29 and 30. The excessively small particle size is unfavorable for the generation of oxygenated products due to the metallization of particles. In addition, the activity of hydrogen evolution reaction will be enhanced on the surface of catalysts with smaller particle sizes.[3] Neverthless, the selectivity of C_{2+} alcohols are gradually increased on gas diffusion electrode loaded with larger size particles, which benefits from the stability of low-coordinated Cu/Cu_2O. However, the selectivity of C_{2+} products steep decline owing to the aggregation of nanoparticles. In brief, the appropriate particle size is contributed to the stabilization of enhanced rectifying interface of low-coordinated Cu_L/Cu_2O Mott−Schottky catalyst.

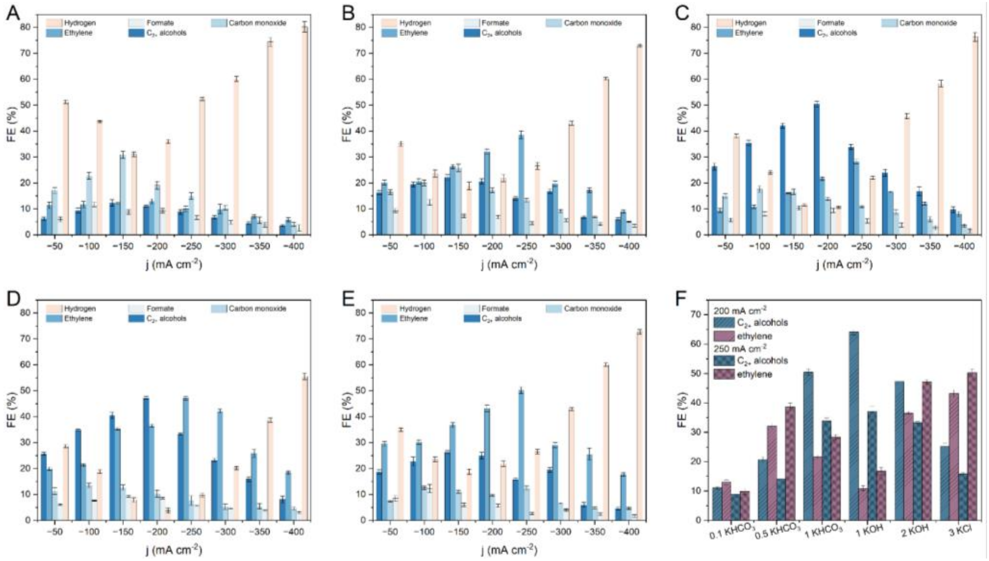

Supplementary Figure 31 | FE of products in 0.1 (A), 0.5 (B), 1 (C) M $KHCO_3$, 2 M KOH (D) and 3 M KCl (E) electrolyte, and (F) statistical bar chart of C_{2+} alcohols and ethylene for Cu_L/Cu_2O. Error bars show the standard deviations calculated from three independent experiments.

As the pH of electrolyte increases, the FE of C_{2+} products on Cu_L/Cu_2O catalyst gains a climb up to ~90%, where the selectivity of C_{2+} alcohols reaches a maximum value in 1 M KOH and decline slightly in more basic electrolyte. In conclusion, it undergoes a electrochemical reduction reaction of CO_2 with synergistic effect of CO_2 coverage and catalyst in pH-compatible electrolyte and thermodynamics-mediated competitive reaction in strong alkaline electrolyte. Besides, the Cl element shows a positive effect for improving the selectivity of C_{2+} products, but the sensitivity of ethylene is more forceful than C_{2+} alcohols.

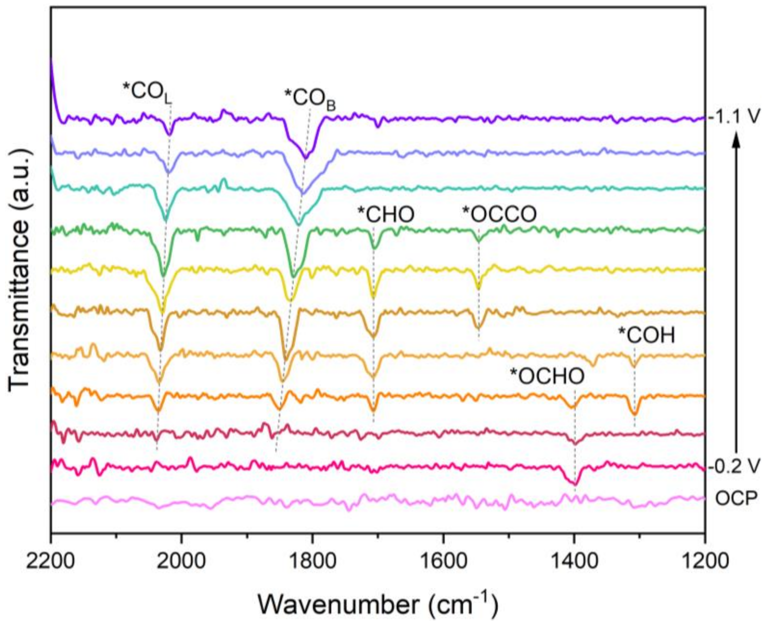

Supplementary Figure 32 | Operando SR-FTIR measurements of Cu_P/Cu_2O with various potentials from -0.2 ~ -1.1 V.

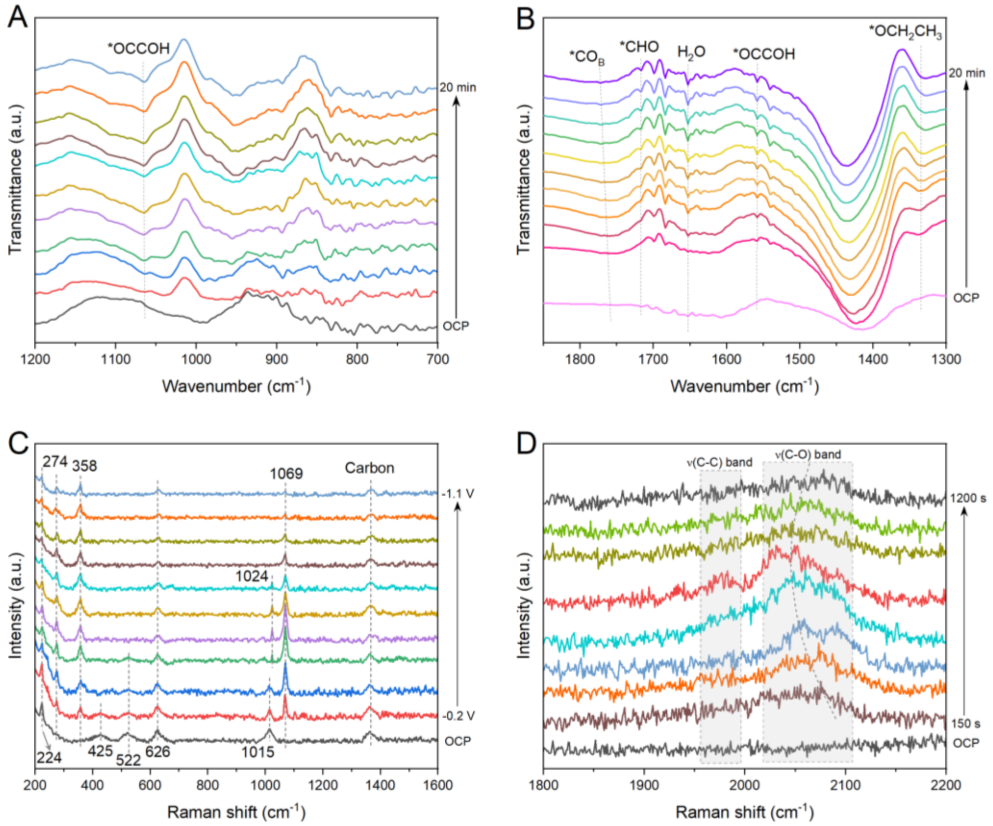

Supplementary Figure 33 | (A and B) Operando SR-FTIR and (C and D) Raman measurements with potentials from -0.2 ~ -1.1 V and time during 20 min at -0.6 V vs. RHE for Cu_L/Cu_2O.

For the Raman spectra of Cu_2O in Supplementary Figure 33, the Raman peaks of Cl-Cu_2O precursor assigned at 224, 425, 522, and 626 cm^{-1}, which are attributed to the $2\Gamma^{-}_{12}$, $4\Gamma^{-}_{12}$, Γ^{+}_{25}, and $\Gamma^{-}_{12} + \Gamma^{+}_{25}$ phonon modes. Group-theoretical analysis predicts that phonons with symmetry Γ^{-}_{12} can assist in the dipole excitation of the 1s exciton. Experimentally, it was found that the phonon-assisted absorption edge of Cu_2O was dominated by the contribution of the Γ^{-}_{12} phonon. Since a Γ^{-}_{12} phonon-assisted photoexcitation of the 1s exciton and radiative recombination of the exciton, the $2\Gamma^{-}_{12}$ mode showed the strongest enhancement and abruptly over the entire tuning range of potentials, where the absorption edge is derived from the photoexcitation of the 1s yellow exciton with emission of Γ^{-}_{12} phonons. In addition, the Raman signals of some silent modes will be enhanced due to nonstoichiometry or impurities. Thus, $\Gamma^{-}_{12} + \Gamma^{+}_{25}$ phonon mode also presents a recognizable signal value owing to the atoms defect of low-coordinated Cu_L/Cu_2O.

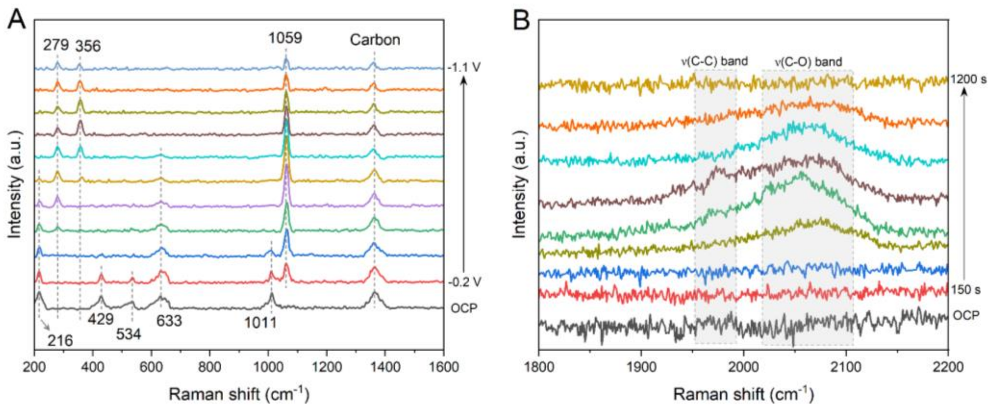

Supplementary Figure 34 | Operando Raman measurements. (A) Various potentials from -0.2 ~ -1.1 V and (B) time during 20 min at -0.6 V vs. RHE of Cu_P/Cu_2O.

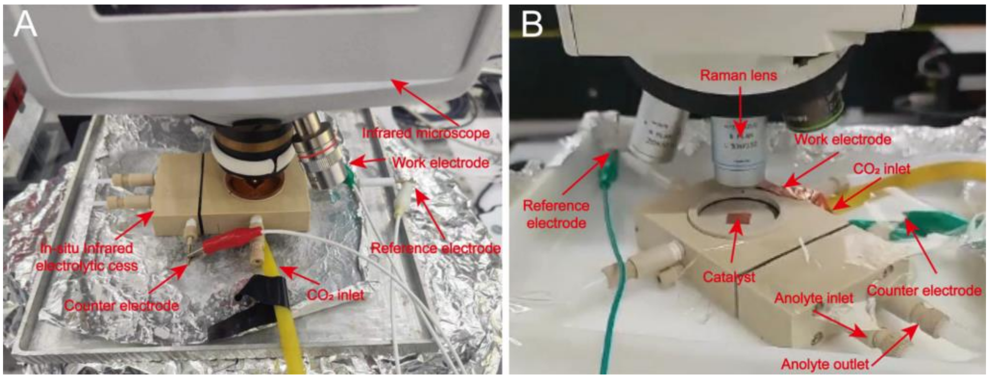

Supplementary Figure 35 | The optical images of in-situ FTIR (A) and Raman (B) tests.

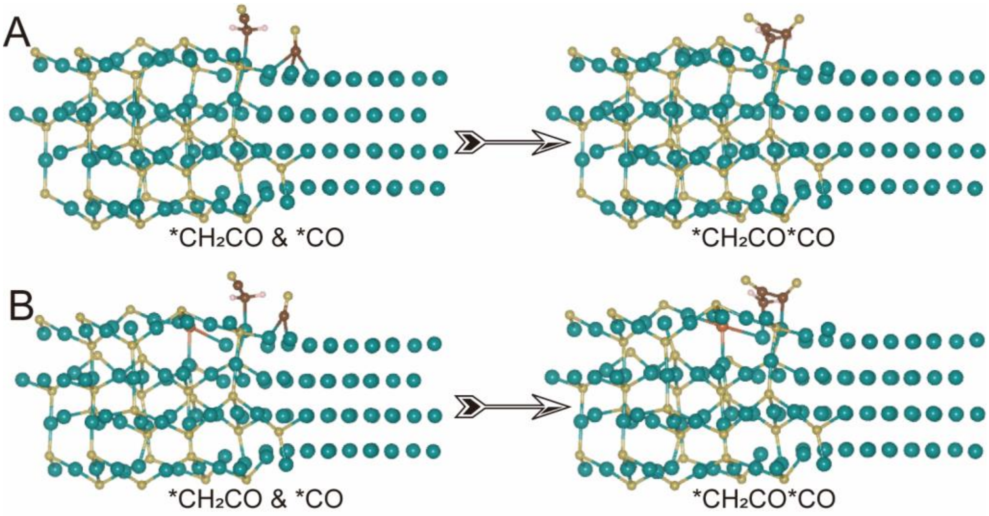

Supplementary Figure 36 | The adsorption models of $*CH_2CO\&*CO$, * CH_2CO*CO intermidates on Cu_P/Cu_2O (A) and Cu_L/Cu_2O (B).

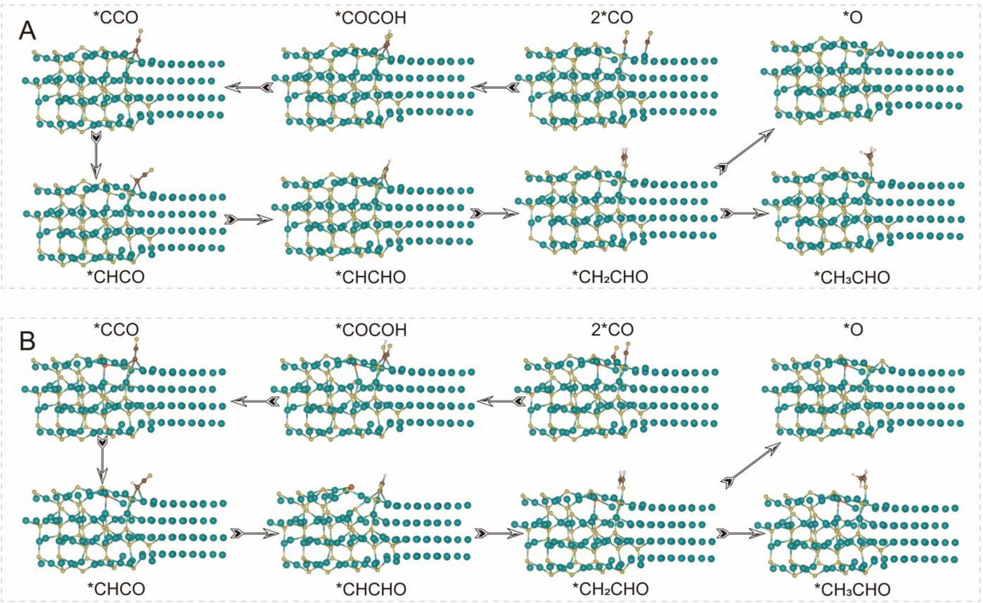

Supplementary Figure 37 | The adsorption models of 2*CO, *COCOH, *CCO, *CHCO, *CHCHO, *CH_2CHO, *CH_3CHO and *O intermidates on Cu_P/Cu_2O (A) and Cu_L/Cu_2O (B).

Supplementary Table 1. Crystallographic parameters of Cl-Cu_2O obained from XRD Rietveld refinement.

**Space group**<br><br>**atoms**	**Pn-3m (No. 224)**<br><br>**site**	**x**	**y**	**z**	**occ.**
Cu	4b	0	0	0	1
O	2a	0.25	0.25	0.25	0.7503
Cl	2a	0.25	0.25	0.25	0.2398
a = 4.2756 Å	c = 4.2756 Å	V = 78.16 Å^3	R_{wp}=8.7%	R_p=13.6%	gof=1.92

Supplementary Table 2. The surface composition of samples based on the conclusion of XPS.

**element**	**O (at%)**	**Cu (at%)**	**Cl (at%)**
Cu_2O	38.94	61.06	-
Cl-Cu_2O	24.57	72.14	3.29
Cl-Cu_2O After Ar^+ etching-30 nm	22.76	73.8	3.43
Cl-Cu_2O After Ar^+ etching-60 nm	21.3	75.06	3.64
NH_4Cl-Cu_2O	72.16	19.8	8.04
NH_4Cl-Cu_2O After Ar^+ etching-30 nm	15.84	83.23	0.93
Cu_P/Cu_2O	15.2	84.8	-
Cu_L/Cu_2O	120.84	78.22	1.14
Cu_L/Cu_2O After Ar^+ etching-30 nm	21.46	77.05	1.49
Cu_L/Cu_2O After Ar^+ etching-60 nm	22.14	76.22	1.64

Supplementary Table 3. The value of adsorption energy for intermediates on Cu, Cu_2O, Cu_P/Cu_2O and Cu_L/Cu_2O.

△G (eV) Catalysts / Intermediates	Cu	Cu_2O	Cu_P/Cu_2O	Cu_L/Cu_2O
*CO	-0.95997	-1.30467	-0.77464	-1.22519
2*CO	-1.83383	-2.68048	-1.77312	-2.31417
3*CO	-2.7229	-4.06014	-2.8268	-3.33948
4*CO	-3.57177	-5.44489	-3.75751	-4.3442
*CH_2CO	-0.5828	-1.08177	-0.46675	-0.99172
*CH_2CHO	-2.1442	-1.80451	-2.53158	-2.73368

Supplementary Table 4. EXAFS fitting results at Cu K−edge for Cu_L/Cu_2O.

	shell	CN	R(Å)	$σ^2$	$ΔE_0$	R factor
Cu foil	Cu-Cu	12	2.54±0.01	0.0088	5.1±0.5	0.0034
Cu_L/Cu_2O	Cu-O	1.7±0.1	1.87±0.01	0.0045	9.5±0.7	0.0025
	Cu-Cu	0.8±0.1	2.56±0.01	0.0046		
	Cu-Cu1	6.0±0.5	3.04±0.01	0.0185		
	Cu-O1	1.8±0.4	3.55±0.02	0.0033		
Cu_2O	Cu-O	1.9±0.1	1.86±0.01	0.0053	6.5±0.6	0.0036
	Cu-Cu	6.8±0.5	3.00±0.01	0.0191		
	Cu-Cu1	3.6±0.5	3.50±0.02	0.0106		

Supplementary Table 5. A comparison of C_{2+} alcohols on various electrocatalysts.

Catalysts	Electrolyzer	Electrolytes	Potential (V vs. RHE)	j_{total} ($mA·cm^{-2}$)	$j_{ethanol}$ ($mA·cm^{-2}$)	$EE_{alcohols}$	Reference
Cu_L/Cu_2O	Flow cell	1 M KOH	-0.66	-200	-113	39.32	This work
K-F-Cu-CO_2	Flow cell	1 M KOH	-0.53	-800	-423	36.45	Adv. Mater. 2022, 34, 2204476
BaO/Cu	Flow cell	1 M KOH	-0.75	-400	-204	35.06	Nat. Catal. 2022, 5, 1081–1088
Cu-DS	H-cell	0.1 M $KHCO_3$	-1.08	-32	-16	34.96	Joule 2021, 5, 429–440
	Flow cell	1 M KOH	-0.95	-200	-50	34.97	
R-Cu-C	Flow cell	1 M KOH	-0.8	-47.4	15.4	33.18	Chem Catal. 2023, 3, 100512
N-C/Cu	Flow cell	1 M KOH	-0.68	-300	-156	31.86	Nat. Energy 2020, 5, 478–486
NGQ/Cu-nr	Flow cell	1 M KOH	-0.9	-281.2	-126.5	27.8	Angew. Chem. Int. Ed. 2020, 59, 16459–16464
CuZn alloy	Flow cell	1 M KOH	-0.68	-200	-84	27.43	Angew. Chem. Int. Ed. 2019, 58, 15036–15040
wr-Cu	Flow cell	1 M KOH	-0.91	-800	-328	26.43	Chem. Sci. 2023, 14, 310–316
FeTPP[Cl]/Cu	Flow cell	1 M $KHCO_3$	-0.82	-302	-124	24.56	Nat. Catal. 2020, 3, 75–82
Hex-2Cu-O	H-cell	0.1 M $KHCO_3$	-1.2	-8.5	-2.76	23.76	Nat. Commun. 2022, 13, 5122
	Flow cell	1 M KOH	-0.66	-200	-26	23.25	
Cu-CuI	Flow cell	1 M KOH	-1	-900	-261	18.35	Angew. Chem. Int. Ed. 2021, 60, 14329–14333
$Cu(OH)_2$-D	Flow cell	1 M KOH	-0.54	-250	-42.5	17.99	Angew. Chem. Int. Ed. 2021, 60, 4879–4885
$Li_{2-x}CuO_2$-10	Flow cell	1 M KOH	-0.85	-220	-59.4	16.21	Small 2022, 18, 2106433
F-Cu	Flow cell	2.5 M KOH	-0.54	-400	-68	12.86	Nat. Catal. 2020, 3, 478–487
Cu_2S-Cu-V	Flow cell	1 M KOH	-0.95	-400	-92	9.45	Nat. Catal. 2018, 1, 421–428
Cu-CIPH	Flow cell	7 M KOH	-0.91	-1170	269.1	7.3	Science 2020, 367, 661–666
Cu/NPC-800	H-cell	0.2 M $KHCO_3$	-1.05	-12.13	8.2	36.61	Green Chem. 2020, 22, 71–84
Cu_2O–ZnO	H-cell	0.5 M $KHCO_3$	-0.9	-	-	31.47	J. Power Sources, 2023, 556, 232468
Cu_3Ag_1	H-cell	0.5 M $KHCO_3$	-0.95	-39.7	-17.2	27.64	Adv. Energy Mater. 2020, 10, 2001987
CeO_2−Cu	H-cell	0.1 M $KHCO_3$	-0.6	45.3	13.14	20.45	ACS Materials Lett. 2022, 4, 1999−2008

Cu_I	H-cell	0.1 M $KHCO_3$	-0.9	-38	-10.5	19.76	Angew. Chem. Int. Ed. 2019, 58, 17047–17053
CuO-ZnO_{10}	H-cell	0.5 M $KHCO_3$	-0.8	-5	-3.6	16.26	Electrochimica Acta 2021, 392, 138988
od-Pd_9Cu_{91}	H-cell	0.5 M $KHCO_3$	-0.65	-8.4	-1.3	12.54	Green Chem. 2020, 22, 6497–6509

Supplementary Table 6. The value of reaction energy for C_3 intermediates on Cu_P/Cu_2O and Cu_L/Cu_2O.

Intermediates \ △G (eV) \ Catalysts	**Cu_P/Cu_2O**	**Cu_L/Cu_2O**
*CH_2CO&*CO	0	0
*CH_2COCO	-0.0475	-0.148

Supplementary Table 7. The value of reaction energy for C_2 intermediates on Cu_P/Cu_2O and Cu_L/Cu_2O.

Intermediates \ △G (eV) \ Catalysts	**Cu_P/Cu_2O**	**Cu_L/Cu_2O**
2*CO	0	0
*COCOH	1.78	1.46
*CCO	0.49	0.185
*CHCO	0.106	-0.139
*CHCHO	-0.0894	-1
*CH_2CHO	0.219	-0.099
*CH_3CHO	-0.154	-0.293
*O	0.522	0.17

Supplementary References

1. C. Zhan et al., Critical Roles of Doping Cl on Cu_2O Nanocrystals for Direct Epoxidation of Propylene by Molecular Oxygen. *J. Am. Chem. Soc.* **142**, 14134-14141 (2020).
2. B. Zhang et al., Work function and band alignment of few-layer violet phosphorene. *J. Mater. Chem. A* **8**, 8586-8592 (2020).
3. R. Reske et al., Particle Size Effects in the Catalytic Electroreduction of CO_2 on Cu Nanoparticles. *J. Am. Chem. Soc.* **136**, 6978-6986 (2014).
