# Supplementary material for: Low-coordinated copper facilitates the *CH2CO affinity at enhanced rectifying interface of Cu/Cu2O for efficient CO2-to-multicarbon alcohols conversion
Source: Nat Commun. 2024 Jun 18;15:5172. doi: 10.1038/s41467-024-49247-4 (PMC11189494; doi:10.1038/s41467-024-49247-4)
Supplement: Supplementary file 3 — Description of Additional Supplementary Files [file 41467_2024_49247_MOESM3_ESM.pdf]

Description of Additional Supplementary Files

Supplementary Data 1: Cu-POSCAR, Cu_2O-POSCAR, Cu_P/Cu_2O-POSCAR, Cu_L/Cu_2O-POSCAR
